# Microtubule-based transport is essential to distribute RNA and nascent protein in skeletal muscle

**DOI:** 10.1038/s41467-021-26383-9

**Published:** 2021-10-27

**Authors:** Lance T. Denes, Chase P. Kelley, Eric T. Wang

**Affiliations:** 1 Department of Molecular Genetics & Microbiology, Center for NeuroGenetics, Genetics Institute, University of Florida, Gainesville, FL USA; 2 Biomedical Sciences Graduate Program, University of Florida, Gainesville, FL USA; 3 Genetics and Genomics Graduate Program, University of Florida, Gainesville, FL USA; 4 Myology Institute, University of Florida, Gainesville, FL USA

**Keywords:** Microtubules, Differentiation, RNA transport, Ribosome

## Abstract

While the importance of RNA localization in highly differentiated cells is well appreciated, basic principles of RNA localization in skeletal muscle remain poorly characterized. Here, we develop a method to detect and quantify single molecule RNA localization patterns in skeletal myofibers, and uncover a critical role for directed transport of RNPs in muscle. We find that RNAs localize and are translated along sarcomere Z-disks, dispersing tens of microns from progenitor nuclei, regardless of encoded protein function. We find that directed transport along the lattice-like microtubule network of myofibers becomes essential to achieve this localization pattern as muscle development progresses; disruption of this network leads to extreme accumulation of RNPs and nascent protein around myonuclei. Our observations suggest that global active RNP transport may be required to distribute RNAs in highly differentiated cells and reveal fundamental mechanisms of gene regulation, with consequences for myopathies caused by perturbations to RNPs or microtubules.

## Abstract (web summary)

It is increasingly recognised that the spatial localisation of RNA is important for proper cellular function. Here, the authors investigate RNA localisation in skeletal muscle and develop methods to show that global active transport of RNA is required to maintain dispersion of gene products in the large muscle syncytium.

## Introduction

Regulated RNA localization is deeply conserved and has been implicated in diverse biological processes ranging from yeast reproduction to memory formation in the mammalian brain^1^. RNAs are localized as ribonucleoprotein (RNP) granules that form via the recruitment of RNA-binding proteins (RBPs) to RNA *cis-*elements, which together dictate the biophysical properties and interacting partners of the RNP^2^. RNA localization has been especially well-studied in neurons, where local synthesis of proteins at synapses is required for proper function^3^. Directed transport is essential to rapidly deliver RNAs to thousands of neuronal synapses, sometimes as far as a meter away from the nucleus^4^. While directed transport of RNAs can also occur in spherical dividing cells, e.g. myosin-dependent transport of *ASH1* mRNA to the bud tip of *S. cerevisiae*^5^, the extreme morphology of neurons engenders a strict requirement for directed mRNA transport. To put this in perspective, it has been estimated that a β-actin mRNA would take 48 days to arrive at the tip of a 500-µm-long axon by diffusion alone^6^.

Similarly to neurons, striated skeletal muscle cells, or myofibers, are large, post-mitotic, and highly differentiated^7^—but in contrast to neurons, they are syncytial tubes containing hundreds of myonuclei^8^. In healthy adult muscle, myonuclei are typically located at the periphery of the myofiber and are evenly spaced apart (except at specialized junctional regions, where they cluster); this configuration is proposed to maximize efficiency of gene expression^9,10^. Myonuclear density varies between muscle types: in mouse extensor digitorum longus, nuclei are spaced ~30 µm apart, and the volume of syncytial cytoplasm per nucleus, often referred to as the “myonuclear domain”, is ~5x the volume of a fibroblast^11,12^. Myofibers must populate a large cytoplasmic space with gene products, but also face the added challenge of coordinating multiple nuclei within a shared cytoplasm. While this problem has been appreciated for decades^13^, the mechanisms by which RNAs from each nucleus distribute within that space are poorly understood. Similarly to neurons, aberrant RNA metabolism also underlies a variety of striated muscle diseases^14–18^, and some RBPs, including TDP-43 and MBNL, are involved in disease pathogenesis in both muscle and neurons^19,20^. Despite a potential role for RNPs to regulate the unique morphological and functional demands of muscle, few investigations have been conducted on RNA localization in myofibers. As a result, we have limited understanding of principles of RNP transport in this tissue and whether they parallel those observed in other cell types.

Various descriptions of RNA localization patterns in muscle cells are have been published, including: (1) enrichment at myotendinous or neuromuscular junctional (NMJ) regions^21,22^, (2) confinement near nuclei^23–25^, (3) enrichment at the myofiber surface^26–28^, (4) uniform dispersion between nuclei and within the myofiber core^29,30^, and (5) association with cytoskeletal filaments or striated patterning^31–33^. Notably, many of these studies were performed in cultured cells and/or employed low-resolution autoradiographic techniques, and modern single molecule fluorescent in situ hybridization (smFISH) techniques have not yet been widely applied to adult myofibers, in part due to high levels of background autofluorescence.

Here, we develop methods to image single RNA molecules together with protein markers in ex vivo skeletal myofibers, and we characterize localization patterns of a diverse set of RNAs. We uncover an intimate relationship between RNAs, sarcomeres, and the microtubule network, and we show that muscle development triggers an absolute reliance on microtubules to disperse mRNAs and avoid accumulation of large RNP granules near myonuclei. We discover that protein synthesis also occurs along cytoskeletal networks, but that efficient RNA transport does not depend on translation. By observing motile RNP granules in live myotubes, we identify diffusive and directed transport states, estimate motion parameters, and confirm by computational simulation that observed RNA localization patterns in myofibers can only be achieved by a significant directed transport component. These observations outline principles of RNA localization in muscle, with broad implications for RNA-protein homeostasis and overall muscle function.

## Results

### RNAs are dispersed throughout skeletal myofibers

**Fig. 1:**
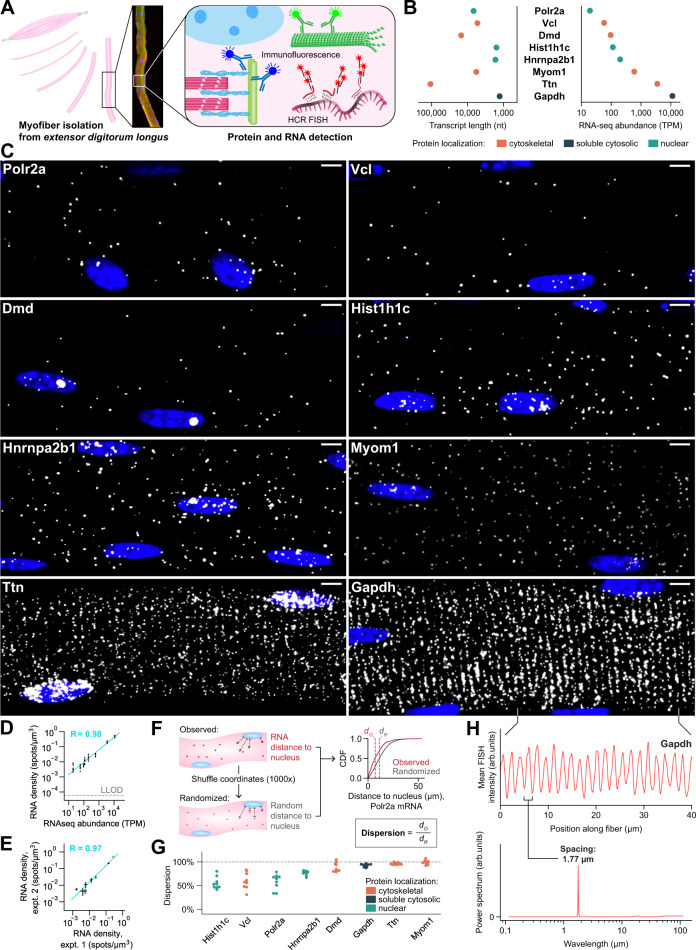
RNAs can be reproducibly and accurately detected in myofibers by HCR FISH and are dispersed in the myofiber cytoplasm. **A** Schematic describing experimental strategy to label RNAs and proteins of interest in adult skeletal muscle. **B** Transcript length (nucleotides, nt) and abundance in *tibialis anterior* muscle (transcripts per million, TPM) for each RNA studied; colors represent encoded protein localization. **C** Representative FISH images for each RNA studied. Scale bars: 5 μm. **D** RNA density (spots/µm^3^) measured from FISH images compared with transcripts per million (TPM) values from a *tibialis anterior* RNAseq dataset. Dotted line is lower limit of detection (LLOD). Trendline: LLS regression; Pearson *R* = 0.98; *p* < 0.05, Wald test. **E** RNA densities compared across separate experiments. Black bars are the mean ± s.d. of RNA density. Experiment 1: *n* = 10 myofibers (Polr2a, Hist1h1c, Ttn, GFP) or *n* = 9 myofibers (Vcl, Dmd, Hnrnpa2b1, Myom1, Gapdh). Experiment 2: *n* = 3 myofibers. Trendline: LLS regression; Pearson *R* = 0.97; *p* < 0.05, Wald test. **F** Schematic describing percent dispersion calculation with example cumulative distribution function (CDF) for Polr2a RNA. **G** Percent dispersion for each RNA studied, points are colored as in Fig. 1B. Dotted line indicates fully uniform dispersion. **H** Mean Gapdh FISH signal intensity of myofiber in **C** plotted for 40 µm along the longitudinal axis (top). The power spectral density of this signal (bottom).

We first developed a platform for robust quantitative analysis of subcellular RNA localization patterns in adult mouse EDL myofibers using a hybridization chain reaction (HCR v3.0) RNA FISH strategy^34^ (Fig. 1A) and a robust computational pipeline for image segmentation (see Methods section and Supplementary Fig. 1A). Using this platform, we studied a diverse set of eight genes to determine whether properties such as gene expression level, transcript length, or encoded protein function were related to mRNA localization patterns (Fig. 1B). This set included mRNAs encoding sarcomere proteins (Ttn, Myom1), costamere proteins (Vcl, Dmd), a nuclear/cytoplasmic shuttling RNA-binding protein (Hnrnpa2b1), a histone protein (Hist1h1c), a subunit of RNA polymerase II (Polr2a), and a metabolic protein (Gapdh). All studied mRNAs are 5’-capped and polyadenylated, including Hist1h1c, which is polyadenylated in differentiated cells^35^.

We observed discrete FISH spots throughout the cytoplasm and nuclei of myofibers for all genes studied (Fig. 1C, Supplementary Movie 1, and Supplementary Table 1). FISH spot densities (spots/μm^3^) were strongly correlated with mRNA copy numbers estimated from RNA-seq (Fig. 1D) and highly reproducible across experiments (Fig. 1E). We used a negative control probe set against GFP mRNA (Fig. 1D) to estimate the lower limit of detection (LLOD) for our method at ~4 TPM (see Methods section). In an effort to determine the number of RNAs contained in each spot, we quantified pixel intensities; however, we did not observe quantal peaks in pixel intensity distributions, as is often observed with standard smFISH approaches, potentially due to variance introduced by the HCR amplification step (Supplementary Fig. 1B)^36^. The number and size of intranuclear spots was variable between genes and may reflect differences in pre-mRNA transcription or processing rates for each gene.

We found that cytoplasmic mRNAs were not confined within individual myonuclear domains, but instead appeared dispersed between myonuclei to varying degrees, indicating that all mRNAs studied frequently travel more than tens of microns from progenitor nuclei. We quantified the percent dispersion of cytoplasmic RNAs relative to myonuclei by comparing observed versus randomized distance to nucleus distributions for each gene (Fig. 1F), and found a shift towards nuclei for all RNAs studied except Myom1 (Fig. 1G, *p* < 0.05 by Two-sided Mann–Whitney *U* test). Despite this shift, all RNAs still readily populated regions of each myofiber furthest from nuclei (Supplementary Fig. 1C). The extent of dispersion was correlated with RNA density but not with transcript length (Supplementary Fig. 1D, E). Interestingly, the variability of both RNA dispersion and RNA density across fibers was greatest for the lowly expressed RNAs, possibly reflecting bursty kinetics of transcription. The extent of dispersion also did not clearly relate to encoded protein function, as the cytoskeletal-protein-encoding Vcl mRNA was among the least dispersed. Finally, all RNAs studied were detected at the myofiber surface and in the core, including Dmd and Vcl, which encode surface-localized proteins, although some RNAs, such as Ttn, Gapdh, and Myom1, were more evenly dispersed within the core than others (Supplementary Fig. 1F). Overall, our observations suggest that, regardless of function and localization of the encoded proteins, RNAs are well dispersed throughout the myofiber syncytium.

### RNAs co-localize with Z-disks and microtubules

We observed that Ttn mRNA in the cytoplasm exhibited a clear striated pattern, consistent with previous observations in cultured myotubes^32^ and cardiomyocytes^37^, but we were surprised to find that Gapdh mRNA was similarly striated. We quantified the periodicity of the Gapdh FISH signal by Fourier analysis, and we found that the peak wavelength of this pattern corresponded to the length of the sarcomere (Fig. 1H). The striated patterns of Gapdh and Ttn mRNAs motivated us to directly assess whether these and other mRNAs might lie along Z-disks, M-lines, or regions in between. Interestingly, microtubules in muscle cells form a distinct perpendicular lattice with bundles leading from myonuclei out to sarcomeres (Supplementary Movie 2)^38^. Due to the distance that RNAs travel from progenitor nuclei and the role of microtubules in transporting RNAs in other contexts, we were also motivated to assess the relationship between RNAs and the microtubule lattice.

**Fig. 2:**
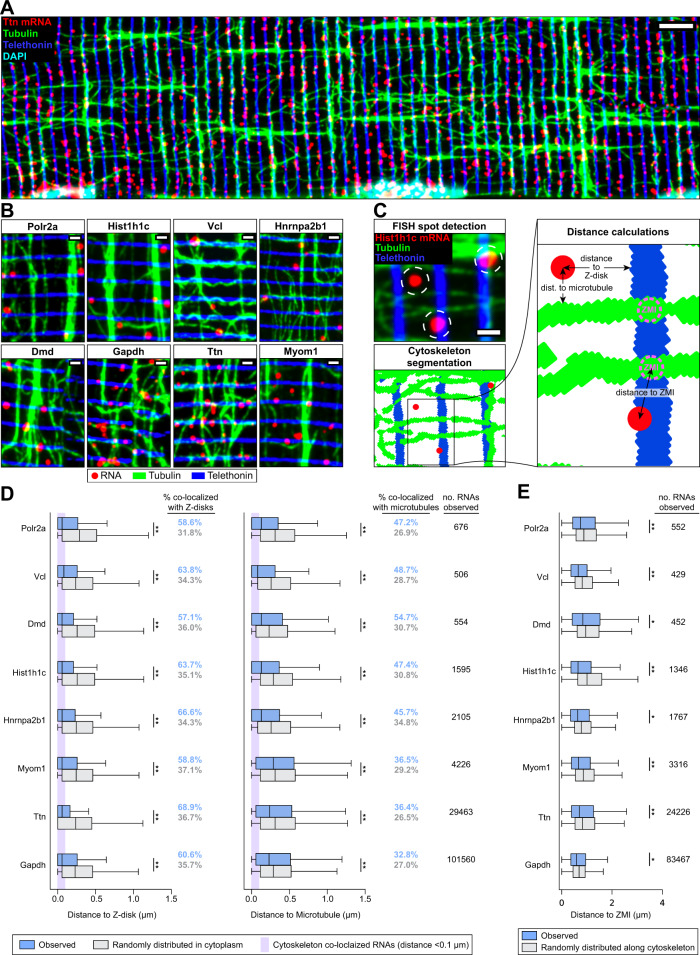
RNAs co-localize with Z-disks and microtubules. **A** Representative image of IF/FISH co-labeling of Ttn RNAs (red), tubulin protein (microtubules, green), and telethonin protein (Z-disks, blue) in an isolated myofiber. Scale bar: 5 µm. **B** Zoomed-in regions of IF/FISH co-labeled myofibers as in **A** for each RNA studied. Scale bars: 1 µm. **C** Schematic describing the computational pipeline used to assess mRNA proximity to cytoskeleton from images of IF/FISH co-labeled myofibers (**A**). **D** Distances from cytoplasmic spots (blue boxes) to Z-disks (left) and microtubules (right) compared to null distributions generated from randomly selected cytoplasmic coordinates (gray boxes). Data from *n* = 10 myofibers (Polr2a, Hist1h1c, Ttn, GFP) or *n* = 9 myofibers (Vcl, Dmd, Hnrnpa2b1, Myom1, Gapdh). Spots located within 2 pixels (~0.1 μm) of either filament were considered “cytoskeleton co-localized” (purple shaded region, percentages). Box plots show minimum, first quartile, median, third quartile, and maximum. *p < 0.03, **p < 10^−4^, Two-sided Mann–Whitney *U* test. **E** Distances from cytoskeleton-associated spots to ZMIs (blue boxes) compared to a null distribution generated from randomly selected coordinates along the cytoskeleton (gray boxes). Number of myofibers same as **D**. Box plots show minimum, first quartile, median, third quartile, and maximum. *p < 0.03, **p < 10^−4^, Two-sided Mann–Whitney *U* test.

We performed IF and FISH to co-label microtubules, Z-disks, and each of the eight mRNAs studied, and we analyzed the localization of each RNA relative to both cytoskeletal filaments (dataset described in Supplementary Table 1). Surprisingly, we found that RNAs from all genes studied were clearly co-localized with both Z-disks and microtubules (Fig. 2A, B). In the absence of sarcomere and microtubule labeling, this localization pattern was visually apparent for highly expressed genes, and co-labeling of protein markers and mRNAs revealed similar co-localization even for lowly expressed genes. We quantified the localization of each RNA relative to cytoskeletal filaments (Fig. 2C) and found that all mRNAs studied were significantly closer than expected to both Z-disks and microtubules (*p* < 0.05 by Two-sided Mann–Whitney *U* test, Fig. 2D). We also noticed a preferential localization of mRNAs near perpendicular Z-disk-microtubule intersections (ZMIs) and found that the localization of “cytoskeleton co-localized” mRNAs (within 2 pixels or ~0.1 µm of either filament) was significantly biased towards ZMIs (*p* < 0.05 by Two-sided Mann–Whitney *U* test, Fig. 2E). Overall, we were surprised to find that Z-disk localization was not limited to mRNAs encoding sarcomere proteins but instead was a general property of all RNAs examined in this study, regardless of encoded protein function or localization. Even Myom1 mRNA, which encodes the M-line-localized protein myomesin-1, was localized to the Z-disk. The localization of RNAs along microtubule bundles could result from direct interactions between mRNAs and filaments or via mutual exclusion into intermyofibrillar space; however, the enrichment of mRNAs at ZMIs suggests that microtubules may facilitate active transport from nuclei to Z-disks.

### NMJ-specific RNAs co-localize with Z-disks and microtubules around the NMJ

**Fig. 3:**
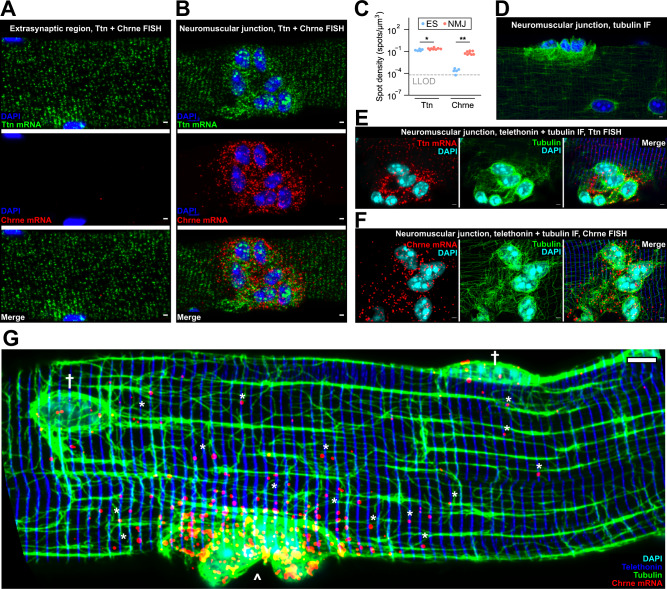
Chrne RNA produced in postsynaptic nuclei is confined to the NMJ region. **A** Representative image of FISH for Ttn (green) and Chrne (red) RNA in an extrasynaptic region of an isolated EDL myofiber. Scale bars: 2 µm. **B** Representative image of FISH for Ttn (green) and Chrne (red) RNA in the NMJ of an isolated EDL myofiber. Scale bars: 2 µm. **C** Quantification of RNA density for Ttn and Chrne RNAs in extrasynaptic (blue) and NMJ regions (red) of myofibers. Extrasynaptic regions: *n* = 5 myofibers; NMJ regions: *n* = 8 myofibers. *p < 0.05, **p < 10^−4^. Two-sided Mann–Whitney *U* test. Dotted line indicates lower limit of detection (LLOD). **D** Tubulin IF in NMJ region of an isolated myofiber. Scale bars: 2 µm. **E** IF/FISH co-labeling of Ttn RNA (red), tubulin protein (microtubules, green), and telethonin protein (Z-disks, blue) at the NMJ of an isolated myofiber. Scale bar: 2 µm. **F** IF/FISH co-labeling of Chrne RNA (red), tubulin protein (microtubules, green), and telethonin protein (Z-disks, blue) at the NMJ of an isolated myofiber. Scale bar: 2 µm. **G** Large field of view image of IF/FISH co-labeling of Chrne RNA (red), tubulin protein (microtubules, green), and telethonin protein (Z-disks, blue). * indicates Chrne RNAs in the extrasynaptic myofiber neighboring the NMJ. † indicates extrasynaptic nuclei containing Chrne mRNAs. ^ indicates NMJ region containing high concentration of Chrne RNA. Scale bar: 10 µm.

The eight mRNAs studied above are transcribed in myonuclei throughout the entire myofiber syncytium. At the NMJ, a cluster of specialized myonuclei at the postsynaptic membrane transcribes a distinct set of mRNAs under control of NMJ-specific transcription factors^39^. We compared localization patterns of NMJ-expressed Chrne mRNA to other mRNAs expressed in the extrasynaptic myofiber. We performed FISH against Ttn and Chrne mRNAs and analyzed their patterns in extrasynaptic and NMJ regions (Fig. 3A, B). As expected, Chrne mRNA was highly concentrated in the immediate vicinity of the NMJ and present in postsynaptic nuclei (Fig. 3B). Expression of Chrne mRNA in extrasynaptic regions was near the LLOD, but was >250-fold higher in the NMJ region (*p* < 0.05, Two-sided Mann–Whitney *U* test, Fig. 3C). Interestingly, Ttn mRNA was also present in postsynaptic nuclei and concentrated near the NMJ, showing a modest increase (1.5-fold; *p* < 0.05, Two-sided Mann–Whitney *U* test) relative to extrasynaptic regions (Fig. 3A–C). However, due to nuclear clustering at the NMJ, these regions contained more nuclei than extrasynaptic regions. Upon normalizing by density of nuclei, we found that Ttn mRNA abundance in the NMJ region was ~50% of that in extrasynaptic regions (*p* < 0.05 by Two-sided Mann–Whitney *U* test), while Chrne mRNA was still >100-fold enriched (*p* < 0.05, Two-sided Mann–Whitney *U* test). These results suggest that postsynaptic nuclei do express sarcomere genes such as Ttn, albeit at lower levels, potentially due to differences in transcriptional and/or post-transcriptional regulation.

The confinement of transcripts around postsynaptic nuclei contrasted with the dispersion of mRNAs observed around extrasynaptic nuclei. Postsynaptic microtubules were much denser and arranged in a nest-like matrix distinct from the grid-like organization of extrasynaptic microtubules (Fig. 3D). We performed IF/HCR FISH co-labeling of microtubules, Z-disks, and Chrne or Ttn mRNA in isolated myofibers and analyzed NMJ regions imaged axially at 100x magnification. mRNAs from both genes were clearly concentrated in the region surrounding the NMJ containing nest-like microtubules, but appeared dispersed and co-localized with Z-disks in neighboring regions containing grid-like microtubules (Fig. 3E, F). Interestingly, this pattern was not appreciably different for Ttn versus Chrne mRNA. When we analyzed NMJ-adjacent regions of myofibers at lower magnification, Chrne mRNAs showed a pattern similar to that observed for the other eight mRNAs studied—dispersed and co-localized with Z-disks and microtubules near ZMIs (Fig. 3G). We also observed RNAs within extrasynaptic, NMJ-adjacent nuclei, suggesting the possibility of low-level extrasynaptic expression of Chrne (Fig. 3G, †).

These data suggest that nest-like microtubules around NMJs might restrict mobility of RNAs produced in postsynaptic nuclei, whereas grid-like microtubules in the extrasynaptic region may promote efficient dispersion of RNAs. Outside the NMJ region, Chrne mRNA co-localizes with Z-disks and microtubules, similar to other extrasynaptic RNAs. Evidence of Chrne transcription in extrasynaptic nuclei also suggests that postsynaptic transcription factors driving Chrne expression (either the proteins or the RNAs that encode them) may travel out of the postsynaptic region to nearby extrasynaptic nuclei.

### RNAs accumulate in the perinuclear region of myofibers after microtubule ablation

**Fig. 4:**
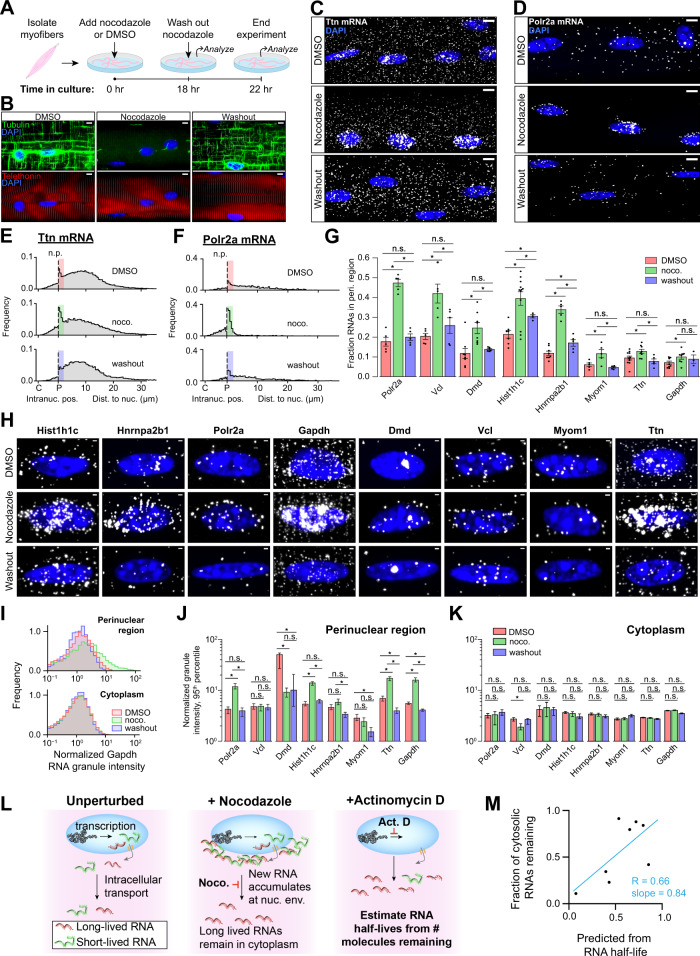
RNAs accumulate in the perinuclear region of myofibers after microtubule ablation. **A** Schematic describing microtubule depolymerization experiment. **B** IF of tubulin (green) and telethonin (red) protein in myofibers from **A**. Scale bars: 5 µm. **C** Representative FISH images of Ttn RNA in myofibers from A. Scale bars: 5 µm. **D** Same as **C** but Polr2a RNA. Scale bars: 5 µm. **E** Intranuclear position relative to centroid (C) and periphery (P) for intranuclear spots and distance to nearest nucleus for cytoplasmic spots; shown for Ttn RNA. Bar colors correspond to treatment condition and shaded area denotes perinuclear region, defined as <2 μm from nuclear periphery (n.p.). Number of myofibers per RNA and condition are Dmd: DMSO-8, nocodazole-8, washout-6; Gapdh: DMSO-9, nocodazole-8, washout-3; Hist1h1c: DMSO-7, nocodazole-12, washout-4; Hnrnpa2b1: DMSO-8, nocodazole-5, washout-5; Myom1: DMSO-5, nocodazole-5, washout-5; Polr2a: DMSO-5, nocodazole-5, washout-5; Ttn: DMSO-11, nocodazole-8, washout-5; Vcl: DMSO-5, nocodazole-5, washout-5. **F** Same as **E** but Polr2a RNA. **G** Fraction of total spots in the perinuclear region for each gene. Colors as in **E**. Bars are mean ± SEM. Black dots are individual images. Number of myofibers as in **E**. *p < 0.05, Two-sided Mann–Whitney *U* test. **H** Representative images for each RNA showing zoomed-in regions of individual nuclei. Scale bars: 1 µm. **I** Cytoplasmic and perinuclear blob intensities for Gapdh RNA in each experimental condition. Colors as in **E**. **J** Intensity of perinuclear blobs. Bars are 95th percentile ±95% CI. n.s. not significant, *p < 0.05, by confidence interval overlap. Confidence intervals were estimated by bootstrapping. **K** Same as **J** but for cytoplasmic blobs. Number of myofibers as in **E**. **L** Schematic describing the effects of Actinomycin D and nocodazole treatments on cytoplasmic RNA concentration. **M** For each RNA, the fraction remaining in the cytoplasm after 18 h nocodazole treatment compared with the fraction predicted by our decay model. Trend line: LLS regression, slope = 0.84; pearson *R* = 0.66; *p* < 0.05, Wald test.

Our observations show that RNAs are frequently dispersed tens of microns away from myonuclei and also co-localize with microtubules and Z-disks near ZMIs in the cytoplasm. Furthermore, RNAs transcribed in postsynaptic nuclei are locally confined in a dense, nest-like microtubule network, but can also disperse and localize along the microtubule lattice if present outside this region. Thus, we hypothesized that microtubules may play a direct role in distributing RNAs from myonuclei. To investigate this possibility, we reversibly depolymerized microtubules by treating cultured myofibers with nocodazole^38^, and we assessed changes to RNA localization patterns by FISH in our eight-gene panel (Fig. 4A, B and Supplementary Table 2).

Strikingly, all genes showed profound accumulation of RNAs around the nuclear envelope after 18 h culture without microtubules, and these accumulations were cleared after microtubule re-polymerization and continued culture (Fig. 4C, D and Supplementary Fig. 3A). We measured the relative position of intranuclear FISH spots and the distance from cytoplasmic spots to the nearest nucleus (Fig. 4E, F and Supplementary Fig. 3A). We then quantified the percentage of RNAs from each gene found in the perinuclear region of each myofiber (within 2 µm of the nuclear periphery) and found a significant increase after microtubule ablation in all cases (*p* < 0.05 by Two-sided Mann–Whitney *U* test) (Fig. 4G). To control for myofiber shape and density of nuclei, we normalized this metric for fiber geometry (see Methods section) and obtained similar results (Supplementary Fig. 3B). After 4 h of nocodazole washout, the perinuclear accumulation of RNAs was no longer detectable for all genes (*p* > 0.05, Two-sided Mann–Whitney *U* test) except for Hist1h1c and Hnrnpa2b1. These two genes showed a significant decrease in the perinuclear fraction between nocodazole and washout conditions (*p* < 0.05, Two-sided Mann–Whitney *U* test), indicating partial clearance. Overall, these observations suggest that microtubules play a critical role in distributing RNAs throughout the cytoplasm in myofibers and that exported RNAs cannot appreciably diffuse away from nuclei after microtubule ablation.

### Perinuclear RNAs gather in large granules after microtubule ablation

We noticed that some RNAs trapped in the perinuclear region upon microtubule depolymerization formed large high-intensity “blobs” as compared to the diffraction-limited spots typically found in the cytoplasm (Fig. 4H). We quantified blob intensities and, using the 95th percentile spot intensity as a metric, we saw that blob intensities were significantly increased in the perinuclear region (but not cytoplasm) following nocodazole treatment (*p* < 0.05 by 95% CI overlap, Fig. 4I–K). After removing nocodazole for 4 h, blob intensities decreased to levels at or below those observed in untreated myofibers. Dmd was a notable outlier: its largest perinuclear granules decreased in intensity in response to nocodazole treatment and remained diminished after washout. Importantly, mRNAs that did not show a significant increase in blob intensity after nocodazole treatment showed instead a significant increase in perinuclear spot number. Conversely, mRNAs showing a modest or insignificant increase in perinuclear spot number did show highly significant increases in blob intensity. Thus, all mRNAs analyzed exhibited accumulation in the perinuclear region of myofibers after nocodazole treatment in some form. Our imaging approach cannot resolve whether high-intensity “blobs” represent higher-order RNP structures containing multiple mRNAs or merely multiple mRNPs in close proximity. Additionally, as shown in our prior analysis, HCR FISH precludes precise determination of the number of mRNAs in each “blob”. Further work and new methods will likely be required to fully address these questions.

### Effects of microtubule depolymerization on nuclear processes

In the nucleus, large FISH blobs at putative transcription sites were diminished following nocodazole treatment. They did not recover during washout, suggesting the potential for negative transcriptional feedback in response to transport blockade (Fig. 4C, H and Supplementary Fig. 3A). We additionally observed accumulation of RNAs at the intranuclear periphery after nocodazole treatment (Fig. 4C–F and Supplementary Fig. 4A). Co-labeling of nuclear pore complex proteins and Ttn mRNA in nocodazole-treated and untreated myofibers revealed accumulation of RNAs on both faces of the nuclear envelope and within nuclear pores, suggesting inhibition of nuclear export (Supplementary Fig. 3C). Together, these observations suggest that nocodazole-induced perinuclear RNA accumulation may cause nuclear export defects and transcriptional inhibition.

### Cytoplasmic RNA depletion following nocodazole treatment is predicted by half-life

While each RNA studied accumulated in the perinuclear region after microtubule depolymerization, the extent of depletion of RNAs from the cytoplasm varied between genes (Fig. 4C–F and Supplementary Fig. 3A). We hypothesized that differential RNA decay rates accounted for these differences (Fig. 4L). To assess this, we estimated half-lives of each RNA after 0, 6, and 22 h of transcriptional inhibition by actinomycin D. We used these half-lives to model cytoplasmic transcript density after 18 h of complete inhibition of perinuclear RNA egress and found that the predictions of this model correlated with observations after culture in nocodazole (Fig. 4M, Pearson *R* = 0.66) (Supplementary Fig. 4A, B). This result suggests that RNAs observed in the cytoplasm after culture in nocodazole were present prior to treatment and did not travel from the nucleus during treatment. Confounding variables influencing measurement of half-lives by actinomycin D^40^ or other factors that affect RNA metabolism may explain why the relationship is not stronger.

Due to its short half-life, nearly all cytoplasmic Polr2a mRNAs decayed during 18 h of nocodazole treatment (Fig. 4D); thus, any molecules observed in the cytoplasm after nocodazole washout were likely to have traveled there from the perinuclear region after microtubules re-polymerized. By partially washing out nocodazole, we allowed a sparse microtubule network to re-polymerize after 18 h nocodazole treatment. We found Polr2a mRNAs in the cytoplasm only at the ends of microtubule filaments connected to nuclei (Supplementary Fig. 4C), suggesting that these are the only routes of escape for mRNAs to leave the perinuclear region. After 30 min, few RNAs were present at the ends of these segments, and substantial perinuclear accumulation remained, suggesting that egress of RNPs along microtubules is a rate-limiting step of directed RNA transport in myofibers.

Interestingly, we also found that RNA density for all genes except Ttn and Myom1 increased during 18 h of myofiber culture, even in the absence of drug treatment (Supplementary Fig. 4D). Expression of Hist1h1c, which exhibited the strongest upregulation, was also notably heterogeneous between nuclei (Supplementary Fig. 3A and Supplementary Movie 3). Similar heterogeneous bursts of transcription following myofiber culture have been observed for Myod1, resulting in localization of mRNA near myonuclei^29^. Compared to freshly isolated fibers, upregulated RNAs in cultured fibers were noticeably enriched at the myofiber surface and closer to nuclei on average. In the cytoplasm surrounding myonuclei in which mRNAs were highly upregulated, mRNAs localized furthest from progenitor nuclei along the longitudinal nuclear poles, from which the most robust microtubule bundles extend (Supplementary Fig. 3A and Supplementary Movie 3). Given these observations, we infer that RNPs depart myonuclei most efficiently along microtubule bundles at the myofiber surface, but require additional time to disperse throughout the fiber if recent bursts of transcription have occurred.

### Muscle maturation restricts microtubule-independent mRNA dispersion

**Fig. 5:**
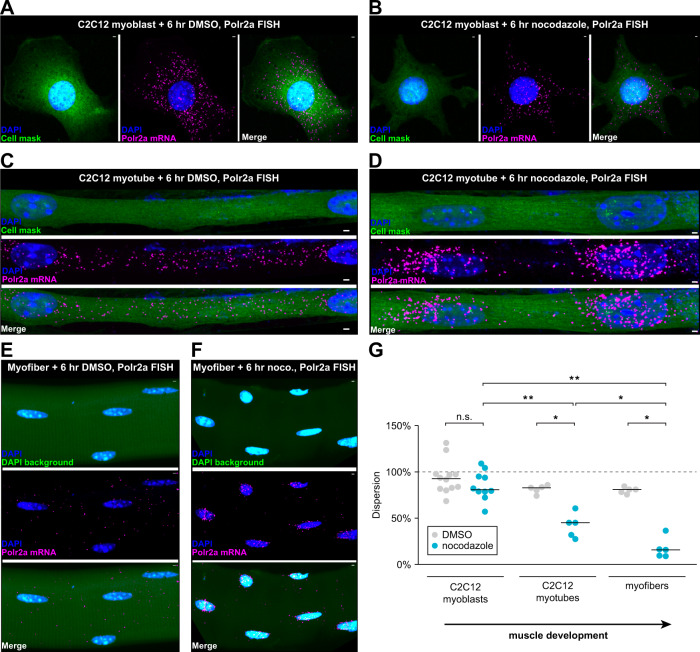
Muscle development restricts microtubule-independent mRNA dispersion. **A** Polr2a FISH in C2C12 myoblast after 6 h control (DMSO) culture. Scale bars: 2 µm. **B** Polr2a FISH in C2C12 myoblast after 6 h culture with nocodazole. Scale bars: 2 µm. **C** Polr2a FISH in C2C12 myotube after 6 h control (DMSO) culture. Scale bars: 2 µm. **D** Polr2a FISH in C2C12 myotube after 6 h culture with nocodazole. Scale bars: 2 µm. **E** Polr2a FISH in myofiber after 6 h control (DMSO) culture. Scale bars: 2 µm. **F** Polr2a FISH in myofiber after 6 hr culture with nocodazole. Scale bars: 2 µm. **G** Percent dispersion for Polr2a RNA in C2C12 myoblasts, C2C12 myotubes, and isolated myofibers treated with nocodazole or control (DMSO) for 6 h. Black lines are medians. *n* = 5 myotubes and myofibers per condition. *n* = 10 and 12 cells for nocodazole and DMSO-treated myoblasts, respectively. *p < 0.05, **p < 0.01, Two-sided Mann–Whitney *U* test.

In adult myofibers, RNAs may depend on microtubules to leave the perinuclear region due to the constraints on diffusion imposed by mature sarcomeres^41^. To study RNA mobility across different stages of sarcomere maturity, we used C2C12 myotubes as a model of muscle differentiation. C2C12 myotubes do not reach the maturity of adult myofibers but do form sarcomeric structures when cultured on patterned hydrogels; thus, they serve as a model for intermediate muscle development^42^. We examined Polr2a mRNA localization after a shorter 6 h nocodazole treatment in C2C12 myoblasts, myotubes, and isolated mouse myofibers (Fig. 5A–F). We measured RNA dispersion in the cytoplasm (see Fig. 1F) in each condition, and found a significant reduction in dispersion in myotubes and myofibers (*p* < 0.05, Two-sided Mann–Whitney *U* test) but not myoblasts (*p* > 0.05, Two-sided Mann–Whitney *U* test) after nocodazole treatment (Fig. 5G). The reduction in myotubes was less dramatic than in myofibers (*p* < 0.05, Two-sided Mann–Whitney *U* test). These observations suggest that microtubule-independent RNA mobility becomes increasingly limited as muscle cells mature.

### Microtubule depolymerization disrupts spatially dispersed protein synthesis

**Fig. 6:**
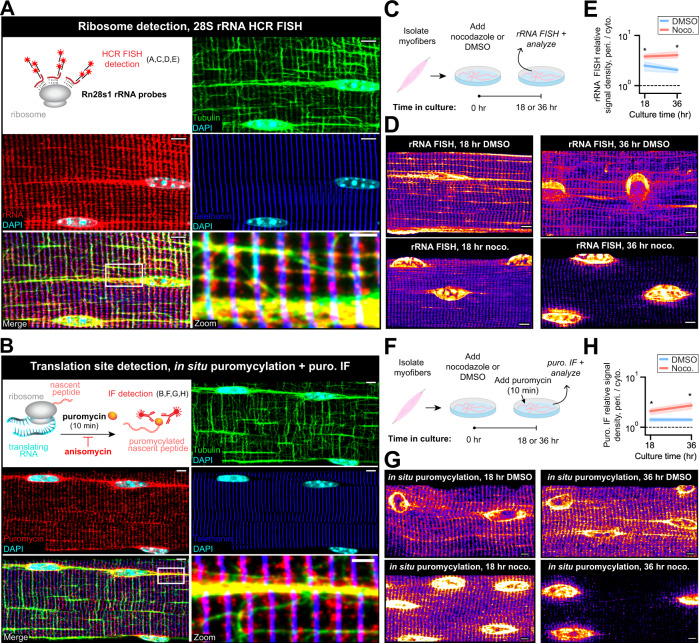
Nocodazole treatment causes accumulation of ribosomes and nascent protein around myonuclei. **A** Ribosomes were detected in isolated myofibers using HCR FISH for 28S ribosomal RNA. Representative images of IF/FISH co-labeling for tubulin (green) and telethonin (blue) proteins with rRNA (red) in myofibers. Scale bars: 5 µm. **B** Active translation was detected in isolated myofibers using in situ puromycylation. Representative images of puromycin detection (red) with tubulin (green) and telethonin (blue) IF in myofibers. Scale bars: 5 µm. **C** Live myofibers were isolated and cultured with nocodazole or DMSO (control) for 18 or 36 hr. At each time point, rRNA FISH was performed. **D** Representative images of rRNA FISH signal in myofibers from **C**. Scale bars: 5 µm. **E** Perinuclear to cytoplasmic relative rRNA signal density (pixel intensity per µm^3^) in myofibers from **C**. Mean ± 95% CI. *n* = 5 myofibers per condition. *p < 0.05, Two-sided Mann–Whitney *U* test. **F** Live myofibers were isolated and cultured with nocodazole or DMSO (control) for 18 or 36 h. At each time point, in situ puromycylation was performed as described in **B**. **G** Representative images of puromycin IF in myofibers from **F**. Scale bars: 5 µm. **H** Perinuclear to cytoplasmic relative puromycin signal density in myofibers from **F**. Mean ± 95% CI. *n* = 5 myofibers per condition. *p < 0.05, Two-sided Mann–Whitney *U* test.

As all mRNAs we studied encode proteins, we investigated the impact of homeostatic and perturbed RNA distribution on protein synthesis. We labeled ribosomal large subunit rRNA via FISH and used in situ puromycylation to label sites of protein synthesis (Fig. 6A, B). Consistent with previous studies in cardiomyocytes, rRNA was localized along Z-disks in unperturbed conditions^37^. We also observed prominent rRNA signal along microtubule bundles and in the perinuclear region (Fig. 6A). Ribosomes outside the perinuclear region were dispersed along the length of the myofiber but highly concentrated at the myofiber surface (Supplementary Fig. 5A). Puromycylation signal was nearly identical to rRNA FISH (Fig. 6B and Supplementary Fig. 5B). Pre-treatment of myofibers with anisomycin (an inhibitor of puromycylation) for 30 min confirmed the specificity of puromycylation (Supplementary Fig. 5C, D). Puromycin signal was more concentrated in the perinuclear region; however, there was more puromycin signal in total outside of this region, indicating that most translation events are occurring along Z-disks in the cytoplasm (Supplementary Fig. 5E).

We also found that the spatial pattern of rRNA and protein synthesis was dependent on microtubules. After 18 h of culture in nocodazole, both rRNA (Fig. 6C–E) and puromycin (Fig. 6F–H) labeling signals were elevated in the perinuclear region and depleted from microtubules relative to control, although enrichment at Z-disks remained. At 36 h, both signals were highly enriched in the perinuclear region and nearly absent from the cytoplasm. The total myofiber puromycylation signal density was reduced at 18 h in nocodazole relative to control but not statistically different at 36 h, and was decreased in control fibers between 18 h and 36 h of culture (Supplementary Fig. 5F). These results suggest that spatially dispersed translation may require microtubules, and that mRNAs trapped in the perinuclear region continue to be translated, albeit at lower levels relative to dispersed mRNAs. The initial persistence of translation in the cytoplasm following 18 h of nocodazole treatment aligns with our earlier observation that many long-lived RNAs are still present in the cytoplasm in significant numbers at that time.

### mRNAs are localized to Z-disks and microtubules independently of translation

It is possible that actively translating ribosomes could recruit mRNAs to the cytoskeleton, or mRNAs could be co-localized with the cytoskeleton independently of translation. To distinguish between these possibilities, we cultured myofibers for 12 h in the presence of puromycin—here for long-term translation inhibition, rather than to label nascent peptides—and we examined resulting localization patterns for Polr2a and Ttn RNAs (Supplementary Fig. 5G, H). RNAs from both genes remained dispersed throughout the myofiber and co-localized with Z-disks and microtubules (Supplementary Fig. 5I, J). These results suggest that while the localization of ribosomes and protein synthesis require microtubules, mRNA localization and co-localization with the cytoskeleton is not dependent on translation.

### Factors regulating translation and transport of RNPs co-accumulate with trapped mRNA

**Fig. 7:**
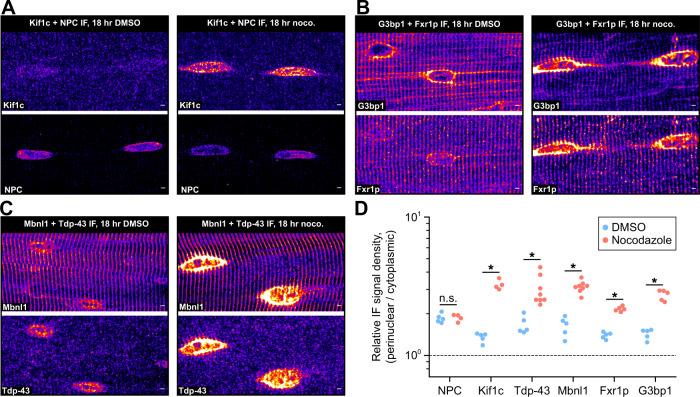
Regulators of RNP granule formation, transport, and translation co-accumulate at the nuclear periphery of nocodazole-treated myofibers. **A** Representative images of Co-IF against the nuclear pore complex (NPC, labeled with mAb 414) and Kif1C in myofibers cultured for 18 h in the presence of nocodazole or control (DMSO). Scale bars: 2 µm. **B** Representative images of Co-IF against G3bp1 and Fxr1p in myofibers cultured for 18 h in the presence of nocodazole or control (DMSO). See also Supplementary Fig. 6A, B. Scale bars: 2 µm. **C** Representative images of Co-IF against the Mbnl1 and Tdp-43 in myofibers cultured for 18 h in the presence of nocodazole or control (DMSO). Scale bars: 2 µm. **D** Perinuclear to cytoplasmic relative IF signal density in nocodazole (red) and control (DMSO, blue) myofibers. *n* = 5 myofibers per protein/condition. *p < 0.05, Two-sided Mann–Whitney *U* test.

Given that RNAs and nascent protein synthesis appear to require microtubules for efficient localization in muscle, we asked whether spatial patterns of motors and RBPs are also dependent on intact microtubules, by staining control and nocodazole-treated myofibers for a variety of factors via IF (Fig. 7A–D). We stained for nuclear pore complex (NPC) proteins as a control, and found that their localization was unaffected by nocodazole. Kif1C is the most highly expressed kinesin motor protein in skeletal muscle tissue and has been shown previously to transport RNPs^43^. We found that Kif1C was diffusely distributed in the cytoplasm of control myofibers and was concentrated in the perinuclear region of nocodazole-treated fibers (Fig. 7A). Fxr1 and G3bp1 are RBPs that regulate translation^44,45^; mutations in *FXR1* in humans are linked to congenital multi-minicore myopathy^46^. We found both proteins to localize along Z-disks and microtubules, and following nocodazole treatment, both accumulated in the perinuclear region and occasionally on Z-disks adjacent to nuclei (Fig. 7B), where we also found RNAs (Supplementary Fig. 6). We found that these populated Z-disks were always connected to nuclei by short, nocodazole-resistant microtubule segments, suggesting some intra- but not inter- Z-disk microtubule-independent RNA mobility.

Tdp-43 and Mbnl1 are RBPs that shuttle between the nucleus and cytoplasm. Both are known to regulate RNA splicing^16,47^ and localization^48,49^, and both are associated with neurological and muscle disease in humans^19,20^. In untreated myofibers, Tdp-43 was enriched in the nucleus, and Mbnl1 showed strong association with Z-disks. These distinct localization patterns suggest differences in the way these RBPs may regulate RNA localization in myofibers. Both RBPs became highly concentrated in the perinuclear region following culture with nocodazole (Fig. 7C). After quantifying average perinuclear and cytoplasmic signal densities, we found that Kif1C and all RBPs visualized were enriched in the perinuclear region upon nocodazole treatment (Fig. 7D, *p* < 0.05, Two-sided Mann–Whitney *U* test). Together, these results illustrate that multiple protein factors involved in mRNP granule formation, transport, and translation co-accumulate with mRNAs in the perinuclear region upon microtubule disruption.

### RNAs exhibit restricted diffusion and directed transport in live myotubes

**Fig. 8:**
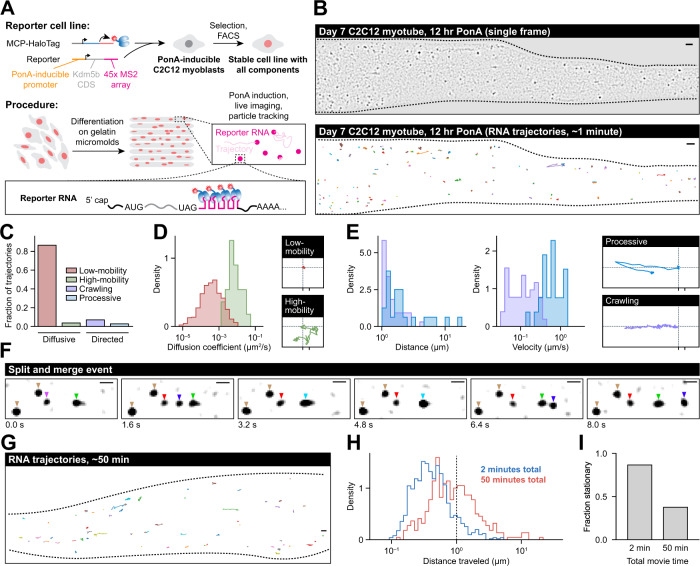
RNAs exhibit restricted diffusion and directed transport in myotubes. **A** Schematic describing MS2 live-cell RNA imaging strategy. **B** Example image of a myotube expressing MS2-labeled RNA (top) and RNA trajectories (bottom) from a 53-s imaging time course. Scale bars: 2 µm. **C** Percentage of RNA trajectories in each motion category. **D** Diffusion coefficients for low-mobility (red) and high-mobility (green) diffusive tracks and example tracks from both groups. X-axis ticks for scale: 0.5 µm. **E** Distance and velocity for processive (blue) and crawling trajectories (purple) along with example tracks from both groups. X-axis ticks for scale: 0.5 µm. **F** Series of images showing RNA particle splitting and merging events. Colored triangles denote particle identities. Scale bars: 1 µm. **G** Example RNA tracks from a myotube expressing MS2-labeled RNA during a 50-min imaging time course. Scale bars: 2 µm. **H** Maximum distance traveled for each track from the 50-min (red) and 2-min (blue) imaging time courses. Tracks below dotted line (1 µm) are categorized as stationary. **I** Fraction of stationary tracks in 50-min and 2-min time courses.

Our observations suggest that mRNAs in myofibers cannot escape progenitor nuclei by passive diffusion; they instead completely depend on microtubules to move throughout the cell. However, we inferred these conclusions via pharmacological inhibition of transport machinery, followed by fixation and imaging, and not by observing RNAs in real-time. To confirm inferences made by analyses in fixed myofibers, we used the MS2 system to fluorescently label mRNAs in live C2C12 myotubes^50^ (Fig. 8A, see Methods section). We chose the Kdm5b coding sequence as our MS2 reporter for basal RNA transport dynamics as it has been previously characterized for live-cell RNA tracking in dividing cells^51–53^ and was observed to display a “non-localized” spatial pattern.

We imaged our MS2 reporter RNA in myotubes continuously, at frame rates >1 per second, to characterize particle motion dynamics (Fig. 8B). We categorized RNA tracks according to their motion parameters into four groups: (1) diffusive, low-mobility; (2) diffusive, high-mobility; (3) directed, crawling; and (4) directed, processive (see Methods section, Fig. 8C and Supplementary Movie 6). We categorized tracks that moved <1 µm as low-mobility; these tracks were mostly stationary but approached diffusion coefficients observed for RNAs in neurons at the high end (max. diffusion coefficient 9.0 × 10^−3^ µm^2^/s, versus 3.8 × 10^−3^ µm^2^/s in neurons), which are roughly an order of magnitude lower than those observed in fibroblasts^54^ (Fig. 8D). We categorized the small subset of RNAs that moved >1 µm in a non-directed fashion as diffusive, high-mobility (Fig. 8D). The most mobile of these particles were nearly as diffusive as RNAs in fibroblasts^54^ (0.04 µm^2^/s, versus 0.09 µm^2^/s in fibroblasts), but they were rare and typically localized in clusters of high-mobility RNAs at the myotube periphery. We believe these are actually regions of under-developed sarcomere structures - an artifact of studying C2C12 myotubes, which are immature relative to adult myofibers. Directed particles moved >1 µm in a single direction and were classified as processive if a run >1 µm occurred without backtracking, or crawling otherwise (Fig. 8E). Processive runs were characteristic of kinesin- or dynein-mediated active RNP transport along microtubules, as has been observed in other cell types^6,54^. Overall run velocity was slightly slower (median 0.63 μm/s) than velocities typically observed in neurons (~1 μm/s), although the fastest runs nearly reached 1.5 μm/s. Most directed runs were short (median 1.6 µm) but the distribution had a long tail with multiple runs >10 µm detected. The crawling motion that we observed is to our knowledge a novel transport state for RNAs. Speeds in this state were slower than processive runs (median 0.11 μm/s), and although the median distance traveled by these particles was similar to median processive run length (1.11 μm), the tail of the distance distribution was much shorter (max. distance 4 μm). We did not observe any instances of directed motion after treating myotubes with nocodazole, confirming these events were microtubule-dependent (Supplementary Movie 7). RNP transport granules have been shown to merge and split in other cell types, and we observed multiple examples of these behaviors in myotubes as well, indicating that these mRNPs can form higher-order granules or complexes (Fig. 8F and Supplementary Movie 8). Together, these data reinforce the conclusions drawn in ex vivo myofibers, that RNA diffusivity is highly restricted in muscle, and that directed transport is the predominant mode of transport.

By imaging with relatively fast frame rates, these particle tracking experiments provided detailed information about the motion of RNAs within a period of 2 min, and showed that most RNAs remain nearly stationary during this time scale. To determine whether RNAs eventually move from these locations, we performed a longer imaging time course in which we captured frames every 15 s for 50 min (Supplementary Movie 9). We tracked 367 individual particles in four movies (Fig. 8G), and found that 141 out of 367 RNAs (38%) still moved <1 μm over the entire 50 min (Fig. 8H, I), indicating that the fraction of particles undergoing motion did not scale linearly with time. This contrasts with observations in neurons, where transport events reposition RNAs at a constant rate^55^. This indicates that some cytoplasmic particles in myotubes may be permanently stationary once anchored, while others are more competent for directed motion.

### Computational simulation demonstrates observed directed transport is necessary and sufficient for RNA dispersion in myofibers

**Fig. 9:**
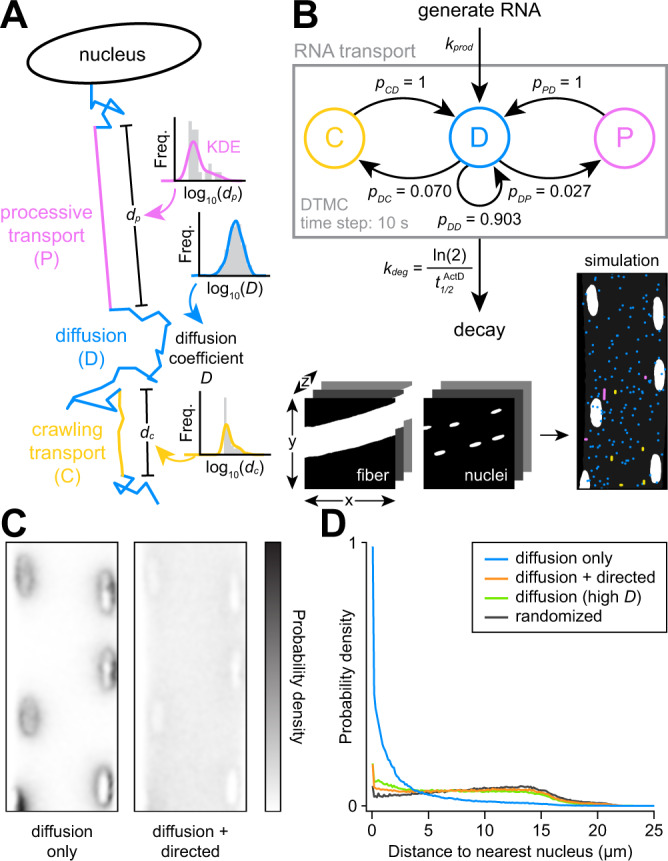
Computational simulation confirms that directed transport is required to disperse mRNA in myofibers. **A** Diagram of RNA mobility states modeled in the simulation of RNA motion in myofibers. **B** Network diagram of the discrete-time Markov chain (DTMC) model developed to simulate RNA generation, motion, and decay. **C** Comparison of RNA localization patterns observed in 1000 h simulations of Polr2a RNA either with or without directed transport states. **D** Distance to nucleus measured for simulated Polr2a RNAs with (orange) or without (blue) directed transport states. Shown for comparison is the distribution from a simulation of Polr2a mRNAs in the high-mobility diffusion state (green) and a null distribution generated from randomly selected cytoplasmic coordinates (gray).

After obtaining quantitative motion parameters from live-cell imaging of C2C12 myotubes, we integrated these measurements into a 3D stochastic simulation of RNA transport in myofibers. In our discrete-time Markov chain (DTMC) model, RNAs are generated at the periphery of myonuclei and decay in the cytoplasm according to their observed copy numbers and half-lives (Fig. 9A). While in the cytoplasm, RNAs transition between diffusive and directed motion states (Fig. 9B, see Methods section). Using fiber and nuclei segmentations obtained from confocal images, we applied this model to simulate RNA transport dynamics within the geometry of real myofibers.

When we simulated Polr2a mRNA but allowed for only low mobility diffusion, we observed accumulation of RNA around nuclei and depletion from nucleus-distal regions. However, by allowing RNAs to transition between low mobility and directed transport states, the RNAs approached full dispersion, mimicking observations of real fixed myofibers at steady state (Fig. 9C, D and Supplementary Movie 10). As compared to a uniformly random null distribution, we still observed slight perinuclear accumulation of RNAs (14% within 2 μm from a nucleus, compared to 8% for randomly distributed RNA), also similar to what we observed experimentally for several genes in fixed myofibers. This likely stems from the requirement that each RNA must first undergo a directed transport event to exit the perinuclear region of the progenitor nucleus.

To determine whether this Markov model accurately describes effects of microtubule depolymerization on RNA transport dynamics, we performed additional simulations in which the available motion states were restricted after reaching steady-state (Supplementary Fig. 7). Simulations of both Polr2a and Myom1 RNAs showed strong agreement between model behavior and images of real myofibers in analogous conditions (Supplementary Fig. 7A–C). However, more Polr2a RNAs were observed in the cytoplasm in the simulated nocodazole condition than in real myofibers, likely due to occasional sampling of high diffusion coefficients for simulated RNAs. Overall, the model appears to effectively describe the RNA transport dynamics of multiple genes with distinct expression levels, decay rates, and responses to microtubule depolymerization.

As a final comparison, we also performed simulations of Polr2a mRNA in the “high-mobility” state (Fig. 9D and Supplementary Movie 10), in which diffusion coefficients are similar to those previously observed in fibroblasts^54^. In this context, we found that the steady-state distribution of RNA indeed approached uniformity (Fig. 9D). Therefore, in principle, if RNPs in myofibers could move as freely as they do in dividing cells in culture, diffusion alone would be sufficient to uniformly populate nucleus-distal regions. Ubiquitous directed transport observed in muscle thus appears to be an adaptation that facilitates efficient distribution of gene cargoes within a dynamic and densely packed syncytium.

This model assumes that RNA diffusion and transport kinetics measured using an MS2-labeled Kdm5b mRNA in C2C12 myotubes are representative of generalized dynamics of mRNAs in myofibers. Different mRNAs will almost certainly display differences in transport and diffusion dynamics. Additionally, the intracellular environment of the mature myofiber is likely different than that of the C2C12 myotube. Nevertheless, this model provides reasonable baseline estimates of RNA dynamics that should be more fully investigated across multiple mRNAs and cellular settings.

## Discussion

Basic principles of mRNA localization are not well understood in skeletal muscle, and it is unknown whether mechanisms operating in other differentiated cell types also apply to muscle syncytia. Here, we developed a method to detect single RNA molecules and protein markers in mature skeletal muscle fibers, and characterized spatial patterns of a diverse panel of RNAs in homeostatic and perturbed conditions. All mRNAs examined are dispersed in the cytoplasm, readily traveling at least tens of microns from progenitor nuclei. We found that enrichment at sarcomere Z-disks is not limited to sarcomere-encoding mRNAs, but instead is a common feature of all mRNAs we studied. Ribosomes and protein synthesis are also localized at this structure, suggesting a role for the Z-disk as a biosynthesis hub, although future work would be required to fully characterize cellular activities occurring in this location. We discovered that muscle development imposes stark restrictions on passive RNA mobility, and that in adult myofibers, microtubule-dependent directed transport appears to be essential for RNAs to travel away from progenitor myonuclei. Disrupting mRNA transport leads to accumulation of large RNP granules and nascent protein in the perinuclear region, with potential implications in muscle disease.

The widespread distribution of RNAs may facilitate optimal distribution of protein products, as well as confer robustness to transcriptional bursting. Interestingly, the extent of dispersion from myonuclei was correlated with expression level and not with transcript length, and was not strictly related to encoded protein function (Fig. 1 and Supplementary Fig. 1). Even exceptionally large mRNAs, such as Ttn, travel efficiently in the myofiber. Additionally, mRNAs encoding nuclear proteins are likely often translated in the vicinity of nuclei from which they did not originate, suggesting a potential mechanism for inter-nuclear gene regulation. Recent studies of myofibers revealed enrichment of some RNAs near myonuclei, and proposed that recent bursts of transcription can lead to this localization pattern^29^. Our observations that low and variable gene expression, as well as culture-induced upregulation, can result in nuclear-proximal localization support this interpretation (Supplementary Movie 3). In contrast to our findings, another recent study found that RNA length, but not expression level, was correlated with dispersion in primary myotubes, with the longest mRNAs being most widely dispersed^56^. This discrepancy may reflect differences in the state of transport machinery and cytoskeletal organization in cultured myotubes as compared to myofibers. Both studies investigated a limited number of RNAs, however, and this question should be addressed in future studies using higher throughput spatial transcriptomics methods.

We were surprised to find that all mRNAs studied are localized along sarcomere Z-disks (Fig. 2 and Supplementary Fig. 2). Decades ago, Z-disk RNA localization was proposed to play a role in sarcomere assembly^57^. However, mRNAs encoding nuclear, metabolic, and even M-line proteins are enriched at Z-disks, together with ribosomes and global protein synthesis machinery (Fig. 6 and Supplementary Fig. 5). These observations evoke a model in which proteins are translated at the Z-disk, yet exhibit sufficient mobility to travel to their final destinations. This would include even proteins resident at the M-line, such as myomesin, according to our data. Studies in cardiomyocytes suggest this model may be broadly applicable to striated muscle cells. For example, Ttn mRNA is localized to Z-disks in cardiomyocytes, but Ttn protein is mobile and likely incorporates into the sarcomere as full-length protein from a soluble pool^37^. Protein synthesis and turnover machinery as well as total polyA mRNA are localized to Z-disks in cardiomyocytes^37,58,59^; thus, this localization pattern appears to be a general feature of striated muscle cell types. Why and how the Z-disk recruits RNAs is not fully clear, but endoplasmic and sarcoplasmic reticular membranes are concentrated at Z-disks and may play a role^60^. Similarly to other cell types, it is possible that actin filaments or actinin itself directly tether RNPs. Alternatively, during muscle contraction, myosin thick filaments may evict RNPs from the A-band region and concentrate them at Z-disks.

RNAs were also co-localized with microtubules and Z-disk-microtubule intersections (ZMIs). When considering the localization of large macromolecular complexes in myofibers, such as RNPs, translating polysomes, or microtubules themselves, mechanical exclusion by the sarcomere may be a driving force. Thus, co-localization of RNPs, translation activity/machinery, and microtubules may be driven by mutual exclusion to the intermyofibrillar space. Mechanical feedback between mitochondria and myofibrils was recently shown to regulate morphogenesis of *Drosophila* muscle^61^; similar mechanical feedback from myofibrils and the concentration of components in the intermyofibrillar space may impact mRNA granulation, transport, and translation. Further studies using super-resolution techniques or advanced electron microscopy may resolve whether these components are indeed exclusively localized in the intermyofibrillar space.

Considering the muscle cytoplasm from this perspective reveals interesting parallels to neurons. Myofibrils run parallel to the long axis of the myofiber and were recently shown to form an interconnected network in skeletal muscle fibers^62^; thus, the intermyofibrillar space can be thought of as a series of long, branched tubules extending throughout the cytoplasm. Examining the dimensions of these “tubules” in comparison to neuronal dendrites reveals a striking similarity: both range from ~100 nm to 1 µm in diameter, if estimating intermyofibrillar space from dimensions of intermyofibrillar mitochondria^63,64^. Thus, in both cell types, RNAs and the components that regulate their localization and translation may be confined to small channels through which they must travel long distances, and in this respect the cytoplasm of these cells may be more similar than widely appreciated. Translation of RNAs along microtubules was also observed, but RNA localization did not depend on translation; further studies would be required to determine whether translation occurs on RNPs in transit.

Completely unexpected was the apparent dependency on microtubules for RNAs to move more than a few microns from the nucleus (Fig. 4). Microtubule dependence for mRNA distribution to the periphery of cultured cardiomyocytes has been observed previously^65^, but we find a much more severe effect in adult skeletal myofibers. Our results agree with a recent study investigating the role of microtubules in distributing RNA in adult cardiac muscle^59^. This study found that microtubule-dependent mRNA dispersion is required to maintain protein synthesis in peripheral regions of myocytes at steady-state and to achieve cardiac growth after the induction of hypertrophy. Following hypertrophy, RNA dispersion in cardiomyocytes appears to be enhanced beyond steady state rates, suggesting that microtubule-dependent transport efficiency can be dynamically altered by external stimuli. These results also point to a role for microtubule-dependent RNA dispersion in skeletal muscle hypertrophy that should be investigated, as there may be important mechanistic differences in how this process is regulated in a highly multinucleated syncytium. Our results and this literature suggest that RNA dispersion increasingly relies on microtubules throughout maturation of cardiac and skeletal muscle, likely due to increasing organization and density of sarcomeres (Fig. 5). Interestingly, we see some evidence for limited mobility within, but not between, Z-disks in the myofiber cytoplasm (Supplementary Fig. 6A, B); however, our data suggests that escape from the perinuclear region is completely dependent on engagement with microtubule-based transport machinery, e.g. kinesins or dyneins. Transport inhibition leads to the formation of large RNP granules, with potential feedback on nuclear export and transcription, highlighting the importance of efficiently clearing RNAs from the perinuclear region (Fig. 4). The grid-like structure of the microtubule lattice in myofibers, with large bundles of microtubules extending from the longitudinal myonuclear poles, likely plays a critical role in moving cargoes quickly from the perinuclear region and distributing them along Z-disks. Indeed, newly produced RNAs move furthest from myonuclei along these large bundles (Supplementary Fig. 3A and Supplementary Movie 3).

Why are RNAs trapped so abruptly in the perinuclear region, and what could underlie export and transcriptional feedback? The linker of nucleoskeleton and cytoskeleton (LINC) complex tethers the nucleoskeleton to the microtubule network^66^, facilitates interactions between the nucleus and molecular motors to position myonuclei^67^, and is involved in mechanosensitive transcriptional regulation^68^ and mRNP export^69^. This complex may play a role in coordinating exported mRNAs for transport and regulating feedback in response to mRNP accumulation. Several myopathies, including Emery-Dreifuss muscular dystrophy^70^ and limb-girdle muscular dystrophy 1B^71^, are caused by mutations to ubiquitously expressed nuclear envelope proteins, highlighting the importance of nuclear envelope function in muscle. Recently, the muscle-specific sk-CIP protein was identified, which regulates myonuclear positioning via interactions with microtubule organizing center (MTOC) proteins and the LINC complex^72^. The unique structure and function of cytoskeletal and nucleoskeletal proteins may regulate or restrict passive RNP egress from the perinuclear region of myofibers.

These findings hold important implications for muscle development, maintenance, and disease. Efficient RNA distribution and protein translation may play roles in facilitating muscle hypertrophy prior to satellite cell recruitment^73^ and in forming new myonuclear domains during regeneration. The strong dependency on directed transport to preserve homeostatic RNP distribution points to vulnerabilities that may underlie myopathies. Amyloid-like “myo-granules” containing TDP-43 form in developing and regenerating muscle, while related TDP-43+ RNP aggregates are observed in muscle diseases such as inclusion body myopathy^74^. *FXR1* mutations have been shown to cause large aggregates of mutated FXR1 protein and polyA RNA in congenital multi-minicore myopathy^46^. In dystrophin-null settings, the microtubule network is highly disorganized and no longer lattice-like^75^. Centralized myonuclei that occur in dystrophic or non-dystrophic settings may lead to inefficiencies in RNP distribution as a result of the sparser microtubule lattice in the myofiber core^76^. The observation that colchicine treatment for gout can occasionally cause myopathy is a long-standing mystery; altered vesicle transport has been proposed to play a role, but our observations suggest a possible contribution from alterations to global RNA and protein distribution^77^.

Finally, observations here may provide broad insights into RNA localization regardless of cell type. RNAs have often been proposed to be either localized or non-localized, with the implication that many non-localized RNAs passively diffuse in the cytoplasm. However, in muscle, it appears that any transit away from the nucleus requires microtubule-dependent transport. Here, the sushi-belt model for RNA distribution^78^ could be the rule rather than the exception—all mRNAs must undergo directed transport by default, with specific localization patterns accomplished via anchoring events. In neurons, RNAs devoid of specific localization elements still undergo directed transport, and it is the directionality and frequency of directed runs that are modulated by specific *cis*-elements^79^. In both of these cell types, diffusion coefficients of mRNAs are orders of magnitude lower than in cultured dividing cells, suggesting that directed transport occurs by default and RNP properties confer specificity. In contrast to neurons, the total fraction of RNPs undergoing directed motion at any given time is much lower in myotubes, in which a significant fraction of RNAs could be permanently immobile (Fig. 6). This may reflect a biological need for RNAs to be stationary at Z-disks for translation, perhaps even for days at a time, given the long half-lives of some transcripts (Supplementary Fig. 5).

Which components of RNPs facilitate directed transport? Effective RNA distribution does not depend on ribosomes (Supplementary Fig. 5), and all mRNAs studied are polyadenylated. Therefore, core machinery such as the cap-binding complex or poly-A binding protein may play important roles, or perhaps multiple RBPs can engage with microtubule motors. Further elucidation of a potential RNA localization code may reveal additional specificities for certain RNPs, with the underlying constraint that, notwithstanding isoform differences, the same RNA sequences must encode localization signals that function properly across numerous cell types and tissues.

## Methods

### Statistics and reproducibility

All experiments were repeated at least three times independently with similar results.

### *Extensor digitorum longus* myofiber isolation

*Extensor digitorum longus* (EDL) muscle was dissected from 10-week-old FVB/NJ mice of both sexes. Mouse maintenance and care followed policies advocated by NRC and PHS publications, and approved by Institutional Animal Care and Use Committee (IACUC), University of Florida. Myofiber isolation followed established methods^80^. Tissues were digested with prefiltered 0.02% collagenase in DMEM at 37 °C for 1 h. Digested EDLs were flushed by pipetting with pre-warmed DMEM in horse serum (HS)-coated plates under microscopy to dissociate individual fibers. Fibers were collected in HS-coated plates containing DMEM and stored in a 37 °C, 5% CO_2_ tissue culture incubator for no more than 1 hr before fixation or continued culture.

### Ex vivo myofiber culture

Isolated myofibers were transferred to HS-coated dishes and cultured in DMEM + 2% HS in a 37 °C, 5% CO_2_ tissue culture incubator for up to 36 h.

### C2C12 mouse myoblasts

The C2C12 mouse myoblast cell line was obtained from ATCC (CRL-1772). Cells were authenticated via morphological assessment. Myogenic potential was assessed by myotube differentiation followed by morphological assessment (see *C2C12 myotube differentiation*). Cells were grown in a 37 °C, 5% CO_2_ tissue culture incubator on tissue-culture-treated dishes in DMEM + 20% fetal bovine serum (FBS) and passaged with trypsin-EDTA before reaching 70% confluency. Original cells from ATCC were expanded and frozen in DMEM + 20% FBS + 10% DMSO. Cells from frozen stocks were used for no more than 5 passages before being discarded to preserve myogenic potential. For FISH, myoblasts were plated on poly-l-lysine coated coverslips (EMS) at 70% confluency and cultured overnight.

### Generation of inducible C2C12 myoblast stable cell lines

Stable ponasterone A (PonA)-inducible C2C12 myoblasts were generated using the PiggyBac transposon system by introducing PB-Neo-pERV3 plasmid and mPB PiggyBac transposon plasmid into C2C12 myoblasts at 50% confluency in six-well plates using Transit-LT1 transfection reagent according to manufacturer’s specifications^81,82^. Cells were cultured for two days and then treated with 200 µg/mL G418 (Geneticin) antibiotic to select for stable cells.

### Generation of C2C12 myoblast cell lines for live-cell RNA tracking

Stable PonA-inducible C2C12 cells were transduced with lentivirus containing an MS2 coat protein HaloTag fusion protein (MCP-Halo) expression cassette. Cells with MCP-Halo integration were isolated by FACS on a Sony SH800 flow cytometer after staining with JF646 HaloTag ligand (Promega). PB-PuroPonA-Kdm5b-MS2 plasmid and mPB PiggyBac transposon plasmid were then introduced to C2C12 myoblasts at 50% confluency in six-well plates using Transit-LT1 transfection reagent according to manufacturer’s specifications. Cells were cultured for two days and then treated with 5 µg/mL puromycin antibiotic to select for stable cells.

### C2C12 myotube differentiation

For differentiation, C2C12 myoblasts were plated at 80% confluency on micromolded gelatin substrates^42^ prepared on glass bottomed dishes or coverslips for live-cell imaging or FISH, respectively. Myoblasts were cultured overnight in DMEM + 20% FBS then switched to DMEM + 2% HS and cultured for an additional 5 days. Media was changed on day 2 and day 4 of differentiation.

### Plasmids and cloning

Plasmid PB-Neo-pERV3 contained bicistronic ecdysone receptor expression cassette from plasmid pERV3 under control of EF1a promoter and PGK-driven neomycin resistance cassette flanked by PiggyBac transposon arms^81,82^. Plasmid was generated using PCR and Clontech In-Fusion cloning according to manufacturer’s specifications. PB-PuroPonA-Kdm5b-MS2 contained KDM5B coding sequence with 45x MS2 hairpins in the 3’ UTR driven by the ecdysone-responsive promoter (EGSH) and a PGK-driven puromycin resistance cassette. Plasmid was generated using Clontech In-Fusion cloning according to manufacturer’s specifications and standard ligation-based molecular cloning. MS2 hairpin array was derived from plasmid kindly gifted by Edouard Bertrand. Kdm5b coding sequence was derived from plasmid kindly gifted by Tim Stasevich. Plasmid used to generate MCP-Halo lentiviral vectors was a gift from Jeffrey Chao (Addgene #64540).

### Antibodies

Polyclonal rabbit anti-Fxr1p (13194-1-AP, Proteintech; dilution: 1:200), polyclonal rabbit anti-KIF1C (ab125903, Abcam; dilution: 1:500), polyclonal rabbit anti-Mbnl1 (kindly gifted by Maury Swanson; dilution: 1:1000), monoclonal rabbit anti-Telethonin (ab133646, Abcam; dilution: 1:1000), monoclonal mouse anti-TDP-43 (ab104223, Abcam; dilution: 1:500), monoclonal mouse anti-G3BP (ab56574, Abcam; dilution: 1:1000), monoclonal mouse anti-Alpha Tubulin (T8203, Sigma; dilution: 1:1000), monoclonal mouse anti-Puromycin (EQ0001, Kerafast; dilution: 1:1000), monoclonal mouse anti-Nuclear Pore Complex Proteins (mAb 414, kindly gifted by Maury Swanson; dilution: 1:1000), and polyclonal chicken anti-Alpha Tubulin (ab89984, abcam; dilution: 1:1000) were used for protein localization via IF, either alone or in combination with FISH.

### Drug treatments

Nocodazole was used at 5 μg/mL in myofibers, myoblasts, and myotubes to depolymerize microtubules. Nocodazole washout to allow microtubule re-polymerization was performed in myofibers subsequent to nocodazole treatment. Myofibers were transferred to a new culture dish containing fresh culture medium (DMEM + 2% HS), and washed three times with fresh culture medium before continued culture. Partial nocodazole washout to allow formation of a sparse microtubule network was performed subsequent to nocodazole treatment by replacing culture medium with fresh medium within the same culture dish. Actinomycin D was used at 5 μg/mL in myofibers to inhibit transcription. Puromycin was used at 100 μM in myofibers to inhibit translation. Ponasterone A was used at 2 µM in stable C2C12 myotubes to induce MS2 RNA reporter expression.

### HCR RNA smFISH and immunofluorescence

HCR v3.0 RNA FISH probes for each gene studied were purchased from Molecular Instruments^34^. Primary probe sets contained between 20 and 30 probes, depending on the length of the mRNA. HCR amplifiers and buffers were purchased from Molecular instruments. Freshly isolated myofibers, ex vivo cultured myofibers, C2C12 myoblasts, and C2C12 myotubes were processed identically. Samples were fixed in 4% paraformaldehyde (PFA) in phosphate-buffered saline (PBS) for 10 min at room temperature (RT), then washed 3 × 5 min with PBS at RT, followed by permeabilization with 1% Triton X-100 in PBS for 10 min at RT. If immunofluorescence was performed, samples were incubated in PBS containing 1% ultra-pure RNase-free BSA (Sigma), 1 U/µL Nxgen RNase inhibitor (Lucigen), and 0.1% Tween 20 (blocking buffer) for 30 min at RT. Blocking buffer was then replaced with blocking buffer containing diluted primary antibody, and samples were incubated for 1 hr at RT followed by 3 × 5 min washes in PBS + 0.1% Tween 20 (PBST). Samples were then incubated in blocking buffer containing diluted secondary antibodies for 30 min at RT and washed 3 ×5 min in PBST. If FISH was performed, samples were then washed once with PBS and fixed again in 4% PFA for 10 min at RT, followed by 3 × 5 min washes with PBS and 1 × 5 mins wash with 2x saline-sodium citrate (SSC) buffer at RT. Samples were then incubated in pre-warmed HCR hybridization buffer (Molecular Instruments) for 30 min at 37 °C in a humidified chamber. During the incubation, primary probes were aliquoted into PCR tubes, heated to 95 °C for 90 s, then diluted to 1 nM in pre-warmed hybridization buffer and kept at 37 °C until hybridization. Hybridization buffer was replaced with hybridization buffer containing diluted probes, and samples were incubated overnight at 37 °C in a humidified chamber. Samples were then washed 5 × 10 min with pre-warmed HCR wash buffer (Molecular Instruments), then washed 2 × 5 min with 5x SSC + 0.1% Tween 20 (SSCT) and incubated in HCR amplification buffer (Molecular Instruments) for 30 min at RT in a humidified chamber. During the incubation, HCR amplifiers were aliquoted into PCR tubes and heated to 95 °C for 90 s, then allowed to cool at RT protected from light for at least 30 min. Amplifiers were diluted to 60 nM in HCR amplification buffer just before amplification. Amplification buffer was replaced with amplification buffer containing diluted amplifiers, and samples were incubated for 3 h at room temperature in a humidified chamber. Samples were then washed 5 × 10 min in SSCT, 1 ×5 min in PBS containing 0.1 µg/mL DAPI, and mounted on slides. This protocol is available at https://protocols.io/file/ef38bni5p.docx

### Puromycylation

Myofibers were treated with puromycin at 2 μM for 10 min to label nascent peptides. Control myofibers were pre-treated for 30 min with 100 μM anisomycin to inhibit puromycylation. Myofibers were washed 3x with pre-warmed PBS and fixed in 4% PFA in PBS for 10 min.

### Fixed sample imaging

All myofiber, myotube, and myoblast fixed sample imaging was carried out on a Zeiss LSM 880 AxioObserver microscope with Airyscan using a Plan-Apochromat 1.3 NA ×40 oil objective (Figs. 1–7, Supplementary Figs. 1–6, and Supplementary Movie 2 and 3), a Plan-Apochromat 1.4 NA ×63 oil objective (Supplementary Movie 1), or a Plan-Apochromat 1.4 NA ×100 oil objective (Fig. 3E, F). Airyscan processing was performed on all images in Zeiss ZEN software. Z-stacks were acquired at 0.5 μm intervals.

### Live-cell imaging

Immediately before live imaging, differentiated myotubes were stained with JF646 HaloTag Ligand (Promega) according to manufacturer specifications. Imaging was performed in Fluorobrite phenol-free culture medium at 37 °C, 5% CO_2_ in a stage mounted incubator using a Zeiss LSM 880 AxioObserver microscope with Plan-Apochromat 1.46 NA ×100 oil objective, widefield fluorescence illumination, and Zeiss AxioCam monochrome camera (Fig. 8). For five fast imaging movies, images were captured continuously for 30 s to 2 min with exposure times between 100 and 500 ms. For long imaging movies, images were captured at 1.0 s exposure time with a 15 s time lag over the course of ~50 min.

### General image analysis pipeline

For each FISH experiment, 3D Airyscan confocal stacks in CZI format were first processed using a general automated pipeline to obtain myofiber and nuclei masks and RNA spot coordinates (Supplementary Fig. 1A). All code is written in Python 3 and is published open source at https://github.com/cpkelley94/muscle-FISH. For each image, voxel dimensions and channel wavelengths were extracted from CZI metadata, and channels were split into 3D arrays. To detect the myofiber geometry, the FISH channel was blurred using a 3D Gaussian kernel, and background signal was binarized using Li’s method for automatic threshold selection^83^. Nuclei were detected by thresholding the DAPI channel using a modified Otsu’s method^84^, followed by contraction of the mask by 0.5 μm in each dimension using morphological erosion. Prior to FISH spot detection, cumulative photobleaching along the z-stack in the FISH channel was corrected by fitting an exponential decay model to the average signal intensity along the z-dimension and multiplying the image by the inverse. Spots were detected using the 3D Laplacian of Gaussian method^85^, with a kernel scale of 1 voxel and a threshold of 2.5%. To robustly control false positive rate, an automatic signal-to-noise filter was applied, which removed spot calls with a maximum intensity lower than the 90th percentile spot intensity divided by the square root of 10. For each image, fiber segmentation, nuclei detection, and FISH spot detection were inspected by a researcher blind to gene identity, and thresholds were manually adjusted when necessary to maintain accuracy.

For each image, FISH spot density (spots/μm^3^) was calculated by dividing the number of detected spots by the total myofiber volume (Fig. 1D, E). The distance from each FISH spot to the nearest nucleus was measured by applying a Euclidean distance transform to the nuclear mask. To generate null distributions for statistical comparison, spot positions were randomized within the cytoplasmic compartment 10,000 times, and the distance to nearest nucleus for each randomized spot was calculated identically as above. The experimental and null distributions were compared using the Two-sided Mann–Whitney *U* test^86^ (Supplementary Fig. 1C). For each FISH image, the dispersion was calculated as the ratio of the median distance to nearest nucleus between the experimental and randomized distributions (Fig. 1F, G), and these ratios were compared across genes to evaluate correlations with mRNA abundance and length (Supplementary Fig. 1D, E)

### Analysis of FISH signal periodicity

The Gapdh FISH image presented in Fig. 1C was rotated to align the striations with the vertical axis, and the mean FISH signal over the z- and vertical axes was plotted for 40 µm along the fiber. The power spectral density was calculated by discrete fast Fourier transform of the signal from the entire fiber image (Fig. 1H).

### Analysis of association of RNAs with cytoskeletal filaments

For each gene, image stacks containing FISH of the RNA of interest and IF of filament proteins were first processed using the general pipeline to generate myofiber and nucleus segmentations and FISH spot coordinates. Microtubule and Z-disk segmentations were generated from IF channels (Tuba1a and Tcap, respectively) using the Allen Cell and Structure Segmenter^87^. Filament segmentations were flattened into 2D by maximum intensity projection along the z-dimension. The 2D distance from each FISH spot to the nearest cytoskeletal filament was measured by applying a Euclidean distance transform to filament segmentations. Spots were considered “cytoskeleton-associated” if they were located within 2 pixels (~0.1 μm) of either filament mask. To determine if FISH spots were located more proximally to Z-disks and microtubules than expected by chance, a null distribution was generated by randomizing spot coordinates in the cytoplasmic compartment. The experimental and null distributions were compared using the one-sided non-parametric Two-sided Mann–Whitney *U* test (Fig. 2D).

Z-disk-microtubule intersections (ZMIs) were detected using a novel approach. Flattened Z-disk and microtubule segmentations were skeletonized using Zhang’s method^88^, and pixels overlapping the nuclear mask were excluded. The two skeletons were merged into a single array with four possible values at each pixel: 0 = background, 1 = Z-disk skeleton, 2 = microtubule skeleton, and 3 = both skeletons. ZMIs were detected by searching the combined mask for a set of small subarrays (motifs) that capture perpendicular intersections. By rotation, reflection, and feature swapping operations, a total of 162 3 × 3 and 4 × 4 motifs were algorithmically enumerated from a set of 14 archetypes. Template matching by fast normalized cross-correlation^89^ was used to efficiently search the combined mask for occurrences of each motif. Multiple intersections called within a radius of 2 px were merged into a single intersection. The distance from each cytoskeleton-associated FISH spot to the nearest ZMI was measured by applying a Euclidean distance transform to the ZMI coordinates. To determine if cytoskeleton-associated FISH spots were located more proximally to cytoskeletal intersections than expected by chance, a null distribution was generated by randomizing spot coordinates in a region of the cytoplasmic compartment within 0.25 μm of either the microtubule or Z-disk skeleton. The experimental and null distributions were compared using the one-sided non-parametric Two-sided Mann–Whitney *U* test (Fig. 2E).

### Analysis of RNA spatial patterns after nocodazole treatment

For FISH spots detected in the cytoplasm, the distance to nearest nucleus was calculated as above. For FISH spots detected inside the nucleus, the intranuclear distance was defined as the relative position of the spot between the centroid and periphery of the nucleus. Intranuclear distance was measured by ray casting, using a 3D surface mesh generated from the nuclear mask to approximate the geometry of the nuclear envelope (Fig. 4E, F and Supplementary Fig. 3A). FISH spots were assigned to spatial compartments (nuclear, perinuclear, cytoplasmic) using the aforementioned feature masks, and the fraction of RNAs within the perinuclear compartment was calculated (Fig. 4G). To normalize the perinuclear fraction metric for invariance to myofiber geometry, we also calculated the perinuclear enrichment as the ratio of the FISH spot densities in the perinuclear and cytoplasmic compartments, presented in Supplementary Fig. 3B. Upregulation of RNAs during culture was assessed by comparing FISH spot density in 18 h DMSO-treated myofibers to spot density in freshly isolated fibers (Supplementary Fig. 4D).

### Estimation of RNA decay rates

For each gene, the mean cytoplasmic FISH spot density was calculated at each time point in the actinomycin D treatment course, and these densities were fit to an exponential decay function using the Levenberg-Marquardt algorithm for nonlinear least squares regression^90^ (Supplementary Fig. 4A, B). RNA half-life was calculated from the optimized decay constant. The ratio of RNAs >5 μm from the nucleus remaining after 18 h treatment with nocodazole was calculated and compared to the expected ratio predicted by exponential decay (Fig. 4M).

### Analysis of perinuclear granule intensity

To enable comparisons of FISH spot intensity across compartments and experimental conditions, spots were first segmented in 3D using the Allen Cell and Structure Segmenter^87^. Briefly, for each image, the FISH channel was smoothed using a 3D Gaussian kernel with a standard deviation of 1 pixel. Spots were detected using the multi-scale Laplacian of Gaussian method with candidate scales of 1 and 2 pixels, and the transform was thresholded to generate a binary mask. To split merged spots, local peaks in FISH intensity were identified and used as seeds for watershed segmentation within the masked area of the image. For each image, segmented FISH spots were called as “cytoplasmic” if the entirety of the binarized blob overlapped with the cytoplasmic mask, and spots were called as “perinuclear” if the blob at least partially overlapped with the perinuclear mask and did not overlap with the cytoplasmic mask. The intensity of each spot was calculated by integrating the raw FISH signal intensity over all voxels in the binarized blob. Within an image, the relative perinuclear spot intensity was defined as the ratio of the raw spot intensity to the median intensity of cytoplasmic spots (Fig. 4I). The five blobs with the highest intensities were dropped for each RNA/location/condition combination to control for false positive detection, and the 95% confidence interval of the 95th percentile spot intensity was estimated by bootstrapping (Fig. 4J, K). Statistical significance at *p* < 0.05 was determined by inspecting overlap of 95% confidence intervals.

### Analysis of RNA dispersion across developmental time course

RNA dispersion was calculated from images of myoblasts, myotubes, and myofibers (Fig. 5) using the general image analysis pipeline to identify RNA spots and segment cells and nuclei. Within each cell type, dispersion was compared between nocodazole and control treatment using the Two-sided Mann–Whitney *U* test. Dispersion after nocodazole treatment was then compared across cell types using the Two-sided Mann–Whitney *U* test.

### Analysis of perinuclear signal density

Images from IF and rRNA FISH nocodazole time course experiments (Figs. 6 and 7) were processed using the general pipeline to segment total myofiber and perinuclear regions. The total signal in the perinuclear and cytoplasmic regions was summed and divided by the total volume of each region to obtain the signal density in each region. All statistical comparisons were made with the Two-sided Mann–Whitney *U* test.

### Analysis of RNA mobility states

For five short imaging (Fig. 8B and Supplementary Movie 4) and five long imaging (Fig. 8G and Supplementary Movie 9) movies of individual myotubes, time series images were cropped and rotated in FIJI such that the longitudinal axis of each myotube was horizontal. Photobleaching was corrected using histogram matching and background was subtracted using a rolling ball radius of 2 pixels. RNA tracks were obtained using the FIJI TrackMate plugin. Quality and size thresholds for spot detection were set manually using histograms. Linking and gap closing distance were set at 0.5 µm and maximum frame gap was set to 3. Tracks obtained for each movie were inspected and manually linked or unlinked as needed (Fig. 8B, G). Trajectories were exported to CSV files and analyzed in Python 3. For each track, spot coordinates were normalized relative to the start position of the track. Then, the maximum distance from the start position to any spot within the track was calculated (Fig. 8H). The percentage of tracks that moved <1 µm (“stationary”) was calculated (Fig. 8I). For five short imaging movies of individual myotubes, tracks were classified into one of four categories based on maximum distance traveled along each axis of the myotube, and the percentage of total tracks in each category was calculated (Fig. 8C and Supplementary Movie 4). Particle trajectories that contained contiguous segments in which a particle moved >1 µm without backtracking were classified as “processive”. The distance from the start and end point of each of these segments was calculated and divided by the elapsed time to obtain the velocity (Fig. 8E). Particle trajectories in which a particle moved at least 1 µm and twice as far in either the X or Y direction were classified as “crawling”. Crawling tracks often moved for only part of the imaging time course and were stationary otherwise. To estimate the velocity of crawling motion, the maximum velocity of any 10 continuous points along crawling tracks was calculated. Maximum distance of the track was used as the distance traveled for “crawling” tracks. Tracks that moved >1 µm and did not travel twice as far in either the X or Y direction were classified as “high mobility” diffusive. Tracks that moved <1 µm were classified as “low mobility” diffusive. For both high and low mobility tracks, mean squared displacement (MSD) was calculated at increasing time lags and a linear least squares regression was performed at the first seven time lags. The slope of the regression line was used to calculate the diffusion coefficient for each particle (Fig. 8D).

### Markov simulation of RNA transport in myofibers

A discrete-time Markov chain (DTMC) stochastic model was developed in Python and applied to simulate RNA localization dynamics in real myofiber geometries with and without active transport states. Fiber and nuclei masks were segmented from 3D Airyscan microscopy images, and these masks were subtracted to define the cytoplasmic space within which RNAs were allowed to move. During the simulation, RNAs were randomly generated at the periphery of nuclei, with an equal probability of spawning from each nucleus. Once generated, RNAs moved within the cytoplasm according to a set of motion states until degradation (Fig. 9A and Supplementary Movie 10). RNA lifetimes were modeled as exponential particle decay with degradation rate calculated from observed half-life measured using actinomycin D (Supplementary Fig. 4B). Production of RNAs was modeled as a Poisson process, and production rate was calculated as the product of degradation rate, fiber volume, and mean cytoplasmic spot density observed in FISH images of myofibers. The time-step *t* for the DTMC was 10 s, approximately equal to the average length of directed transport events observed in live-cell imaging.

While moving in the cytoplasm, RNAs randomly transitioned between available motion states, including diffusion (D), crawling transport (C), and processive transport (P). Transition probabilities were constrained by the average number of RNAs observed in each state during live-cell imaging (Fig. 9B). For each RNA, a diffusion coefficient *D* was selected by sampling from the distribution of “low-mobility” diffusion coefficients measured in C2C12 myotubes, smoothed by kernel density estimation (KDE) (Fig. 8D). During each time-step, positions of RNAs in the diffusion state were translated by a 3D Gaussian random variable with a mean of 0 and a standard deviation of (2*Dt*)^1/2^. If allowed, active transport events lasted for a single time-step, and the distance traveled by directed motion (*d*_*c*_ or *d*_*p*_) was sampled from KDE-smoothed distributions of distances measured in C2C12 myotubes (Fig. 8E). To model the lattice-like structure of microtubules and Z-disks, active transport events were randomly assigned a direction along either the axial or radial dimensions of the fiber, with a 50% probability for each. The axial unit vector was identified by applying a medial axis transform to the fiber mask in 2D and fitting a line to the skeleton by least squares regression. RNAs traveling in the axial direction moved either parallel or antiparallel to the fiber with equal probability. RNAs traveling in the radial direction were allowed to move in any 3D direction perpendicular to the axial unit vector with equal probability. If an RNA exited the allowed cytoplasmic space as a result of its motion during a time-step, the motion event was reverted and sampled again. Particle positions and states were recorded at each time-step for analysis (Fig. 9B).

Using this framework, simulations of Polr2a mRNA transport were conducted for 360,000 time-steps (1000 h) in the following state configurations: (a) D only, (b) D, C, and P. After discarding the initial 10% of the simulation as burn-in to eliminate non-equilibrium effects, samples were taken every 360 time-steps (1 h), and the average distribution of RNA across the fiber was computed across all samples (Fig. 9C).

### Reporting summary

Further information on research design is available in the Nature Research Reporting Summary linked to this article.

## Supplementary information

Supplementary Information

Description of Additional Supplementary Files

Supplementary Movie 1

Supplementary Movie 2

Supplementary Movie 3

Supplementary Movie 4

Supplementary Movie 5

Supplementary Movie 6

Supplementary Movie 7

Supplementary Movie 8

Supplementary Movie 9

Supplementary Movie 10

Reporting Summary

## Data Availability

The source data from this study are provided along with the paper. Raw image files are available upon request. The remaining data are available in the Article and Supplementary Information. Source data are provided with this paper.

## Source data

Source Data

## Code availability

The code developed during this study is available at https://github.com/cpkelley94/muscle-FISH (10.5281/zenodo.5484424).
