# Supplementary material for: Microtubule-based transport is essential to distribute RNA and nascent protein in skeletal muscle
Source: Nat Commun. 2021 Oct 27;12:6079. doi: 10.1038/s41467-021-26383-9 (PMC8551216; doi:10.1038/s41467-021-26383-9)
Supplement: Supplementary file 1 — Supplementary Information [file 41467_2021_26383_MOESM1_ESM.pdf]

SUPPLEMENTARY INFORMATION

Microtubule-based Transport is Essential to Distribute RNA and Nascent Protein in Skeletal Muscle

Lance T. Denes, Chase P. Kelley, Eric T. Wang*

*Address correspondence to eric.t.wang@ufl.edu

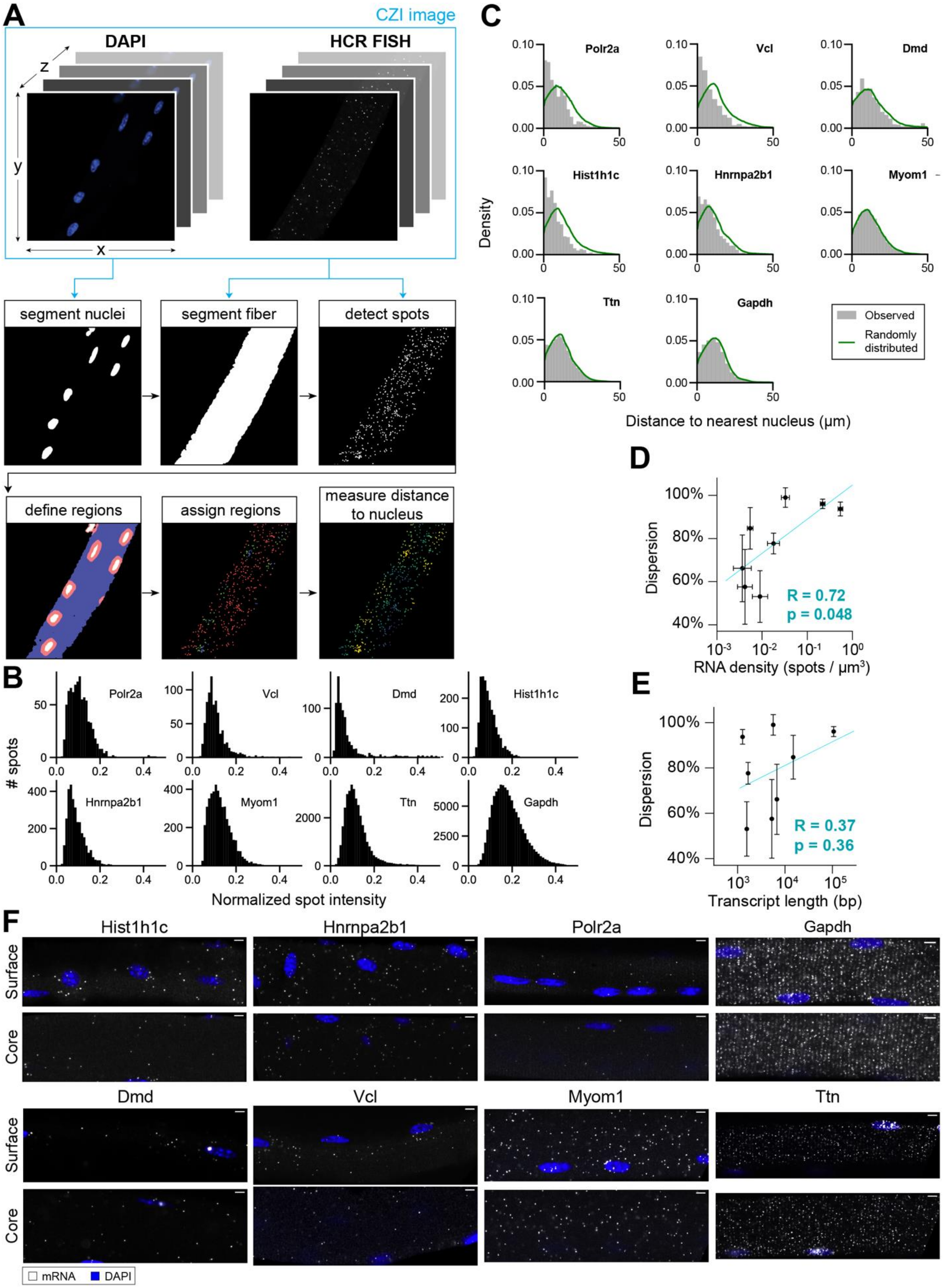

A
CZI image
DAPI
HCR FISH
z
y
x
segment nuclei
segment fiber
detect spots
define regions
assign regions
measure distance to nucleus
B
Polr2a
Vcl
Dmd
Hist1h1c
Hnrnpa2b1
Myom1
Ttn
Gapdh
spots
Normalized spot intensity
C
Density
Observed
Randomly distributed
Distance to nearest nucleus (μm)
D
Dispersion
R = 0.72
p = 0.048
RNA density (spots / μm³)
E
Dispersion
R = 0.37
p = 0.36
Transcript length (bp)
F
Hist1h1c
Hnrnpa2b1
Polr2a
Gapdh
Surface
Core
Dmd
Vcl
Myom1
Ttn
mRNA
DAPI

Supplementary Figure 1. RNAs Can Be Reproducibly and Accurately Detected in Myofibers by HCR FISH and Are Dispersed in the Myofiber Cytoplasm, Related to Figure 1

A) Schematic describing the computational pipeline used to detect RNA FISH spots and segment myofibers and myonuclei. The distance from each FISH spot to the nearest nucleus is measured from coordinates and segmentations.

B) Normalized pixel intensities of FISH spots for each RNA studied. Data from n=10 myofibers (Polr2a, Hist1h1c, Ttn, GFP) or n=9 myofibers (Vcl, Dmd, Hnrnpa2b1, Myom1, Gapdh).

C) Distance from cytoplasmic spots to nearest nucleus (grey bars) compared to a null distribution of randomly selected cytoplasmic coordinates (green lines). Number of myofibers as in (B). *$p<0.05$, Two-sided Mann-Whitney U test.

D) RNA dispersion compared to expression level (RNA density). Number of myofibers as in (B). Mean ± s.d. Trendline: LLS regression. Pearson R=0.72; $p<0.05$, Wald test.

E) RNA dispersion compared to transcript length. Number of myofibers as in (B). Mean ± s.d. Trendline: LLS regression. Pearson R=0.37; $p>0.05$, Wald test.

F) Single optical sections from the surface and core of myofibers labeled by FISH for each RNA studied. Scale bars: 5 µm.

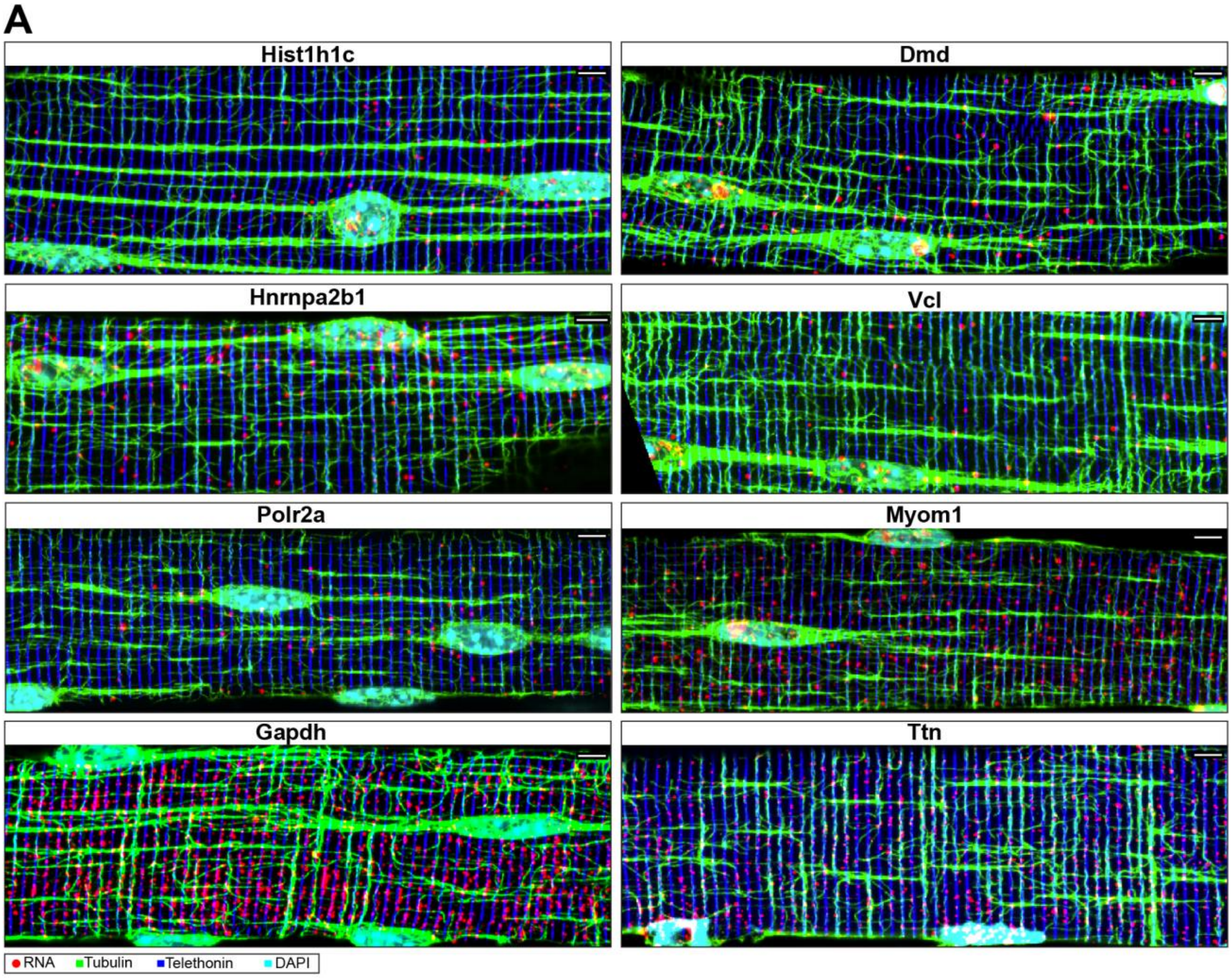

Supplementary Figure 2. RNAs Co-Localize With Z-disks and Microtubules, Related to Figure 2

A) IF/FISH co-labeling of RNAs (red), tubulin protein (microtubules, green) and telethonin protein (Z-disks, blue) in isolated myofibers. Scale bar: 5 µm.

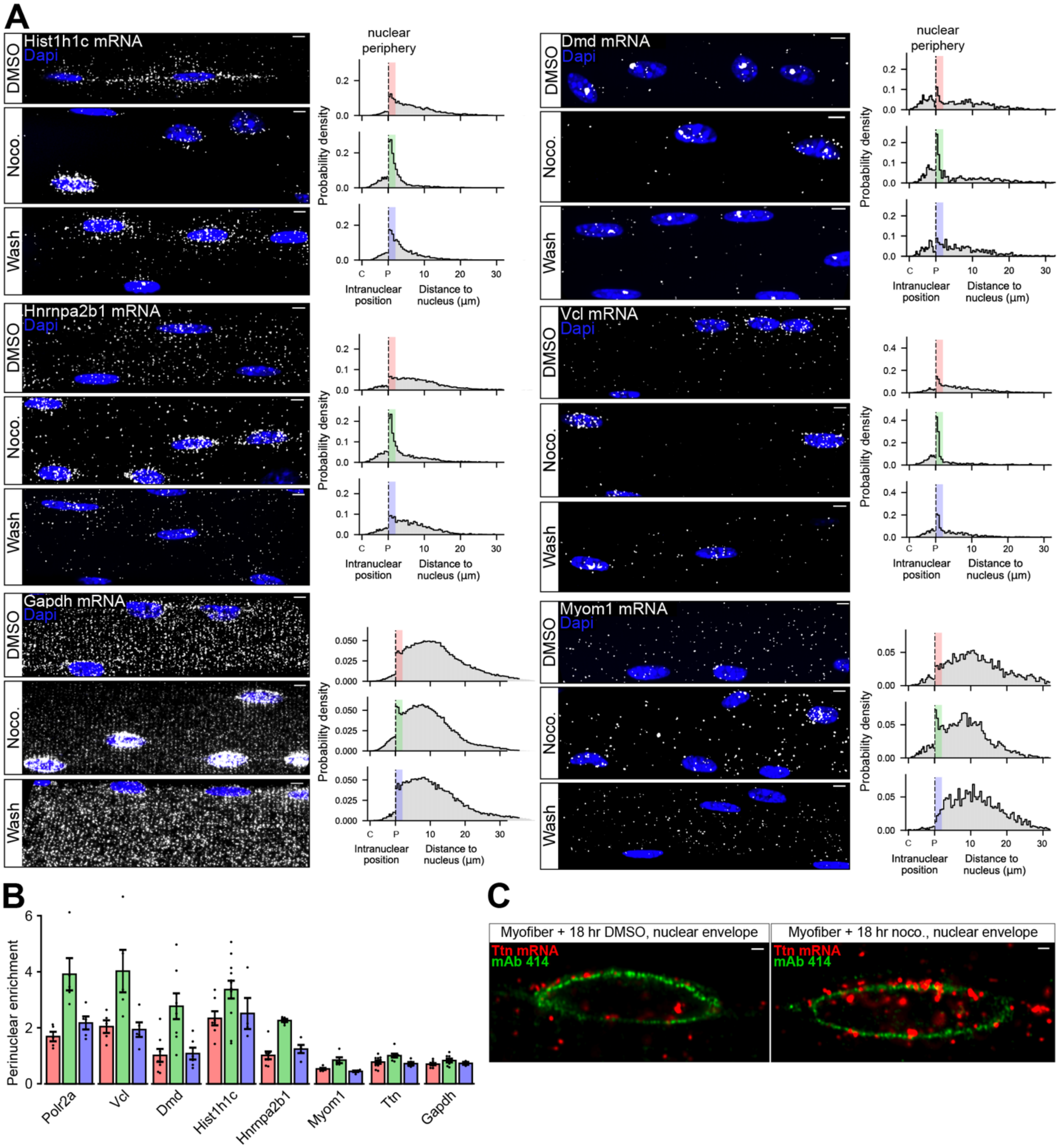
A
Hist1h1c mRNA
Dapi
DMSO
Noco.
Wash
nuclear periphery
Probability density
Intranuclear position
Distance to nucleus (µm)
Hnrnpa2b1 mRNA
Gapdh mRNA
Dmd mRNA
Vcl mRNA
Myom1 mRNA
B
Perinuclear enrichment
Polr2a
Vcl
Dmd
Hist1h1c
Hnrnpa2b1
Myom1
Ttn
Gapdh
C
Myofiber + 18 hr DMSO, nuclear envelope
Myofiber + 18 hr noco., nuclear envelope
Ttn mRNA
mAb 414

Supplementary Figure 3. RNAs Accumulate in the Perinuclear Region of Myofibers After Microtubule Ablation, Related to Figure 4

A) Representative images of FISH for each RNA in myofibers from microtubule depolymerization time course. Scale bars: 5 µm. Histograms show intranuclear position relative to centroid (C) and periphery (P) for intranuclear spots and distance to nearest nucleus for cytoplasmic spots. Bar colors correspond to treatment condition and shaded area denotes perinuclear region, defined as <2 µm from nuclear periphery (n.p.). Number of myofibers per RNA and condition are Dmd: DMSO-8, nocodazole-8, washout-6; Gapdh: DMSO-9, nocodazole-8, washout-3; Hist1h1c: DMSO-7, nocodazole-12, washout-4; Hnrnpa2b1: DMSO-8, nocodazole-5, washout-5; Myom1: DMSO-5, nocodazole-5, washout-5; Polr2a: DMSO-5, nocodazole-5, washout-5; Ttn: DMSO-11, nocodazole-8, washout-5; Vcl: DMSO-5, nocodazole-5, washout-5..

B) Perinuclear enrichment calculated in each image as the ratio of perinuclear spot density to cytoplasmic region spot density. Number of myofibers as in (A). Bars are mean ± SEM. *$p<0.05$, n.s.: $p>0.05$, Two-sided Mann-Whitney U test.

C) Axial optical cross-section through center of myonucleus in myofiber treated with nocodazole (right) or DMSO (left) for 18 hr and co-labeled by IF/FISH for Ttn mRNA (red) and nuclear pore complex proteins (mAb 414, green). Scale bars: 1 µm.

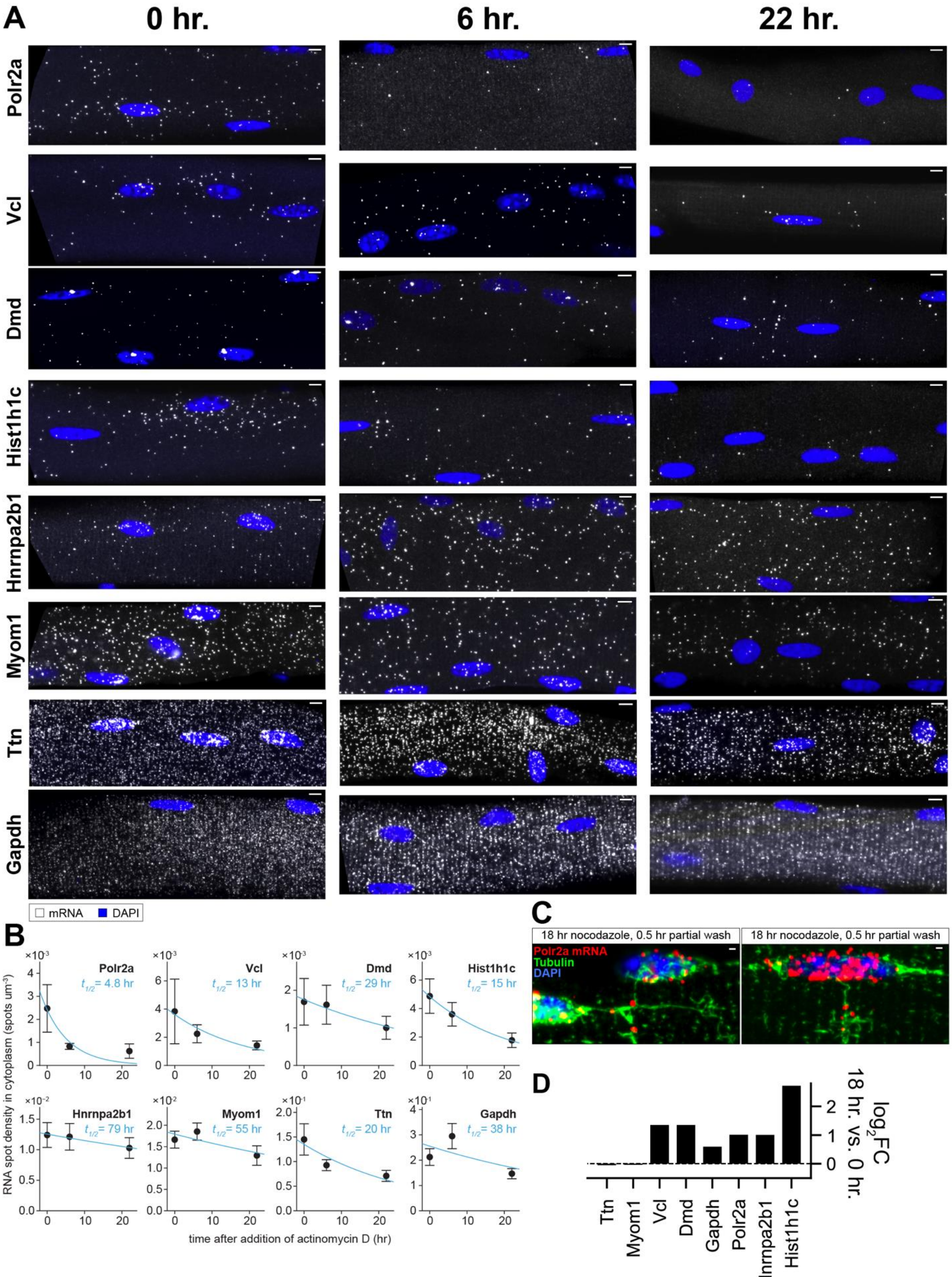
A
0 hr.
6 hr.
22 hr.
Polr2a
Vcl
Dmd
Hist1h1c
Hnrnpa2b1
Myom1
Ttn
Gapdh
mRNA
DAPI
B
RNA spot density in cytoplasm (spots um⁻³)
Polr2a $t_{1/2}$= 4.8 hr
Vcl $t_{1/2}$= 13 hr
Dmd $t_{1/2}$= 29 hr
Hist1h1c $t_{1/2}$= 15 hr
Hnrnpa2b1 $t_{1/2}$= 79 hr
Myom1 $t_{1/2}$= 55 hr
Ttn $t_{1/2}$= 20 hr
Gapdh $t_{1/2}$= 38 hr
time after addition of actinomycin D (hr)
C
18 hr nocodazole, 0.5 hr partial wash
18 hr nocodazole, 0.5 hr partial wash
Polr2a mRNA
Tubulin
DAPI
D
log_2FC 18 hr. vs. 0 hr.
Ttn
Myom1
Vcl
Dmd
Gapdh
Polr2a
Hnrnpa2b1
Hist1h1c

Supplementary Figure 4. RNA Half-lives Predict RNA Distribution After Culture in Nocodazole, Related to Figure 4

A) FISH for each RNA in myofibers treated with actinomycin D for 0, 6, and 22 hr. Scale bars: 5 µm.

B) RNA densities from actinomycin D time course myofiber FISH images. Mean ± SEM. Number of myofibers per RNA/condition are Hnrnpa2b1: 0 hr-6, 6 hr-3, 20 hr-3; Polr2a: 0 hr-3, 6 hr-3, 20 hr-3; Myom1: 0 hr-6, 6 hr-3, 20 hr-3; Gapdh: 0 hr-3, 6 hr-3, 20 hr-3; Hist1h1c: 0 hr-5, 6 hr-3, 20 hr-3; Vcl: 0 hr-5, 6 hr-3, 20 hr-3; Ttn: 0 hr-3, 6 hr-4, 20 hr-3; Dmd: 0 hr-3, 6 hr-3, 20 hr-3. Blue lines are exponential decay curves fit to the data to estimate RNA half-lives ($t_{1/2}$).

C) IF/FISH of tubulin protein (microtubules, green) and Polr2a RNA (red) in myofibers cultured in nocodazole for 18 hr, followed by partial washout and 30 min of additional culture. Scale bars: 1 µm.

D) Log_2 fold-change in RNA density between freshly isolated myofibers and myofibers cultured for 18 hr. Number of myofibers per RNA/condition as in (B).

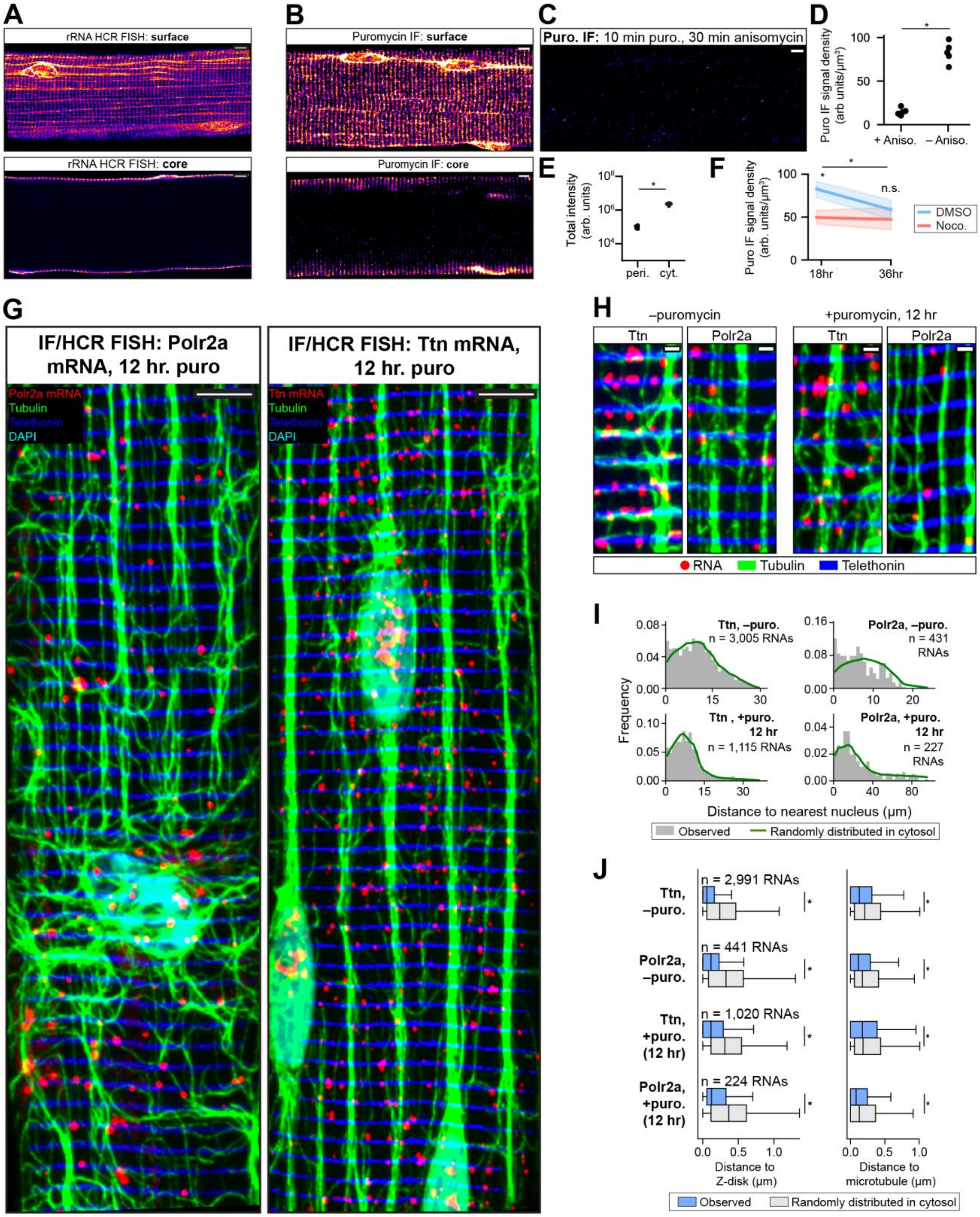

A
rRNA HCR FISH: surface
rRNA HCR FISH: core
B
Puromycin IF: surface
Puromycin IF: core
C
Puro. IF: 10 min puro., 30 min anisomycin
D
Puro IF signal density (arb units/μm³)
+ Aniso.
– Aniso.
E
Total intensity (arb. units)
peri.
cyt.
F
Puro IF signal density (arb. units/μm³)
n.s.
DMSO
Noco.
18hr
36hr
G
IF/HCR FISH: Polr2a mRNA, 12 hr. puro
Polr2a mRNA
Tubulin
Telethonin
DAPI
IF/HCR FISH: Ttn mRNA, 12 hr. puro
Ttn mRNA
Tubulin
Telethonin
DAPI
H
–puromycin
+puromycin, 12 hr
Ttn
Polr2a
RNA
Tubulin
Telethonin
I
Ttn, –puro. n = 3,005 RNAs
Polr2a, –puro. n = 431 RNAs
Ttn , +puro. 12 hr n = 1,115 RNAs
Polr2a, +puro. 12 hr n = 227 RNAs
Frequency
Distance to nearest nucleus (μm)
Observed
Randomly distributed in cytosol
J
Ttn, –puro. n = 2,991 RNAs
Polr2a, –puro. n = 441 RNAs
Ttn, +puro. (12 hr) n = 1,020 RNAs
Polr2a, +puro. (12 hr) n = 224 RNAs
Distance to Z-disk (μm)
Distance to microtubule (μm)
Observed
Randomly distributed in cytosol

Supplementary Figure 5. Nocodazole Treatment Causes Accumulation of Ribosomes and Nascent Protein Around Myonuclei but RNA Is Localized Independently of Translation, Related to Figure 6

A) rRNA FISH in axial optical sections of the surface (top) and core (bottom) of a myofiber. Scale bars: 5 µm.

B) IF against puromycin in axial optical sections of the surface (top) and core (bottom) of a myofiber labeled with puromycin. Scale bars: 5 µm.

C) IF against puromycin in myofiber labeled with puromycin after 30 min anisomycin (inhibitor of puromyclation) pre-treatment. Scale bars: 5 µm.

D) Quantification of total puromycin signal density (pixel intensity per μm^3) with 30 min anisomycin pre-treatment. n=5 myofibers per condition. *$p<0.05$ Two-sided Mann-Whitney U test.

E) Quantification of total puromycin signal in perinuclear and cytoplasmic regions of myofibers. Mean ± 95% CI of n=5 myofibers per condition. *$p<0.05$ Two-sided Mann-Whitney U test.

F) Quantification of total signal density (pixel intensity per μm^3) in myofibers labeled with puromycin after microtubule depolymerization time course. Mean ± 95% CI of n=5 myofibers per condition. *$p<0.05$ Two-sided Mann-Whitney U test.

G) IF/FISH co-labeling of Polr2a (left) or Ttn (right) RNAs (red), telethonin protein (Z-disks, blue), and tubulin protein (microtubules, green) in myofibers treated with 100 µm puromycin for 12 hr to inhibit translation (right). Scale bars: 5 µm.

H) Zoomed regions of images as in G. Scale bars: 1 µm.

I) Distance to nucleus for Ttn and Polr2a RNAs (grey bars) measured in untreated myofibers (from Fig. S1D, left) and in myofibers treated for 12 hr with 100 µM puromycin (right). Distance distributions were compared to null distributions generated from random cytoplasmic coordinates (green). n=3 myofibers per RNA/condition.

J) Distances from cytoplasmic Ttn and Polr2a RNAs (blue boxes) to Z-disks (left) and microtubules (right) in untreated (from Fig. 2D) and puromycin-treated myofibers (bottom), compared to null distributions generated from randomly selected cytoplasmic coordinates (grey boxes). Box plots show minimum, first quartile, median, third quartile, and maximum. n=3 myofibers per RNA/condition. *$p < 0.05$, Two-sided Mann-Whitney U test.

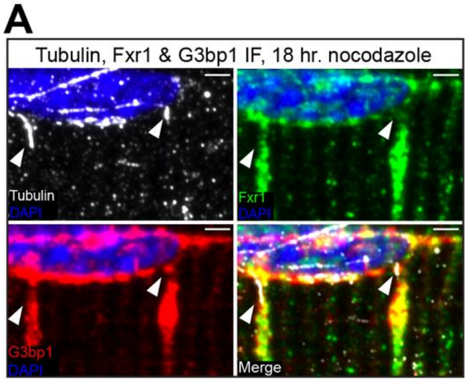

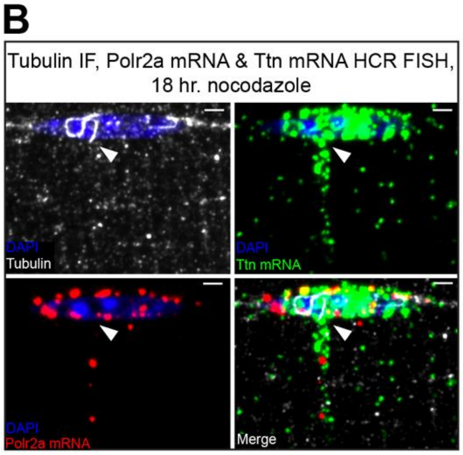

Supplementary Figure 6. RNAs and RBPs Accumulate on Nuclear Adjacent Z-disks, Related to Figure 7

A) IF against G3bp1 (red), Fxr1 (green), and tubulin (microtubules, white) in myofibers treated with nocodazole for 18 hr. Accumulation of both RBPs was observed along Z-disks that contacted nucleus-attached, nocodazole-resistant microtubules (arrowheads). Scale bars: 1 µm.

B) IF/FISH of Polr2a mRNA (red), Ttn mRNA (green), and tubulin protein (microtubules, white) in myofibers treated with nocodazole for 18 hr. Accumulation of both mRNAs was observed along Z-disks that contacted nucleus-attached, nocodazole-resistant microtubules (arrowhead). Scale bars: 1 µm.

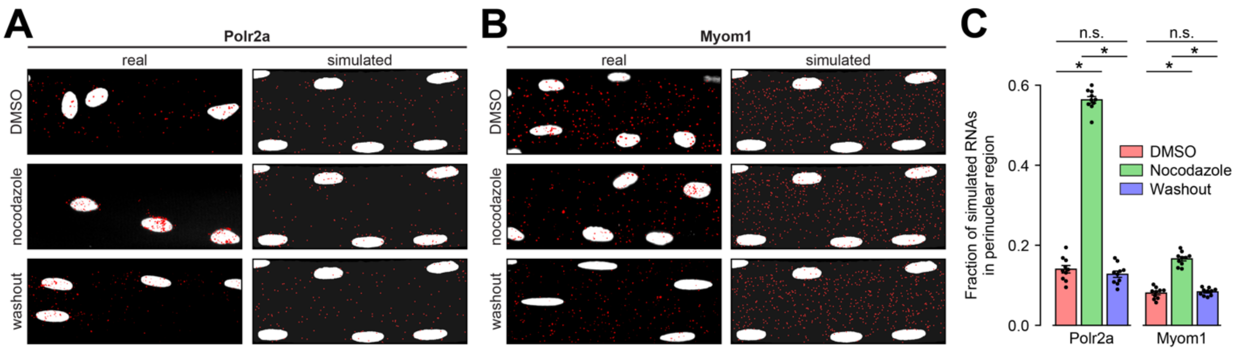

Supplementary Figure 7. Computational Simulation of Microtubule Depolymerization Experiment, Related to Figure 9

A) 2D projections of Polr2a RNA positions (red circles) during simulation of microtubule depolymerization experiment (right), drawn alongside 2D projections of HCR FISH images in analogous conditions (left). RNA transport with D, P, and C states was simulated for 1000 hr to reach steady-state (“DMSO”). The simulation was continued for 18 hr with D state only to model transport in the absence of microtubules (“Nocodazole”), followed by 4 hr with D, P, and C states to model washout of nocodazole and restoration of microtubule-based transport (“Washout”).

B) Same as (A) but Myom1 RNA.

C) Fraction of simulated mRNAs in the perinuclear region after 1000 hr simulation with D, P, and C states (“DMSO”), followed by 18 hr with D state only (“Nocodazole”) and 4 hr with D, P, and C states restored (“Washout”). Bars are mean ± SEM from 10 independent simulations for each gene. *$p < 0.05$, Two-sided Mann-Whitney U test. Compare with Figure 4G.

Supplementary Table 1. Numbers of fibers, nuclei, and FISH spots analyzed in experiments described in Figures 1 and 2.

Gene	n_{fibers}	n_{nuclei}	n_{spots}
Polr2a	10	47	805
Vcl	9	38	691
Dmd	9	35	735
Hist1h1c	10	43	1835
Hnrnpa2b1	9	44	2843
Myom1	9	39	4714
Ttn	10	50	34007
Gapdh	9	43	106046
GFP (neg. ctrl.)	10	49	13

Supplementary Table 2. Numbers of fibers, nuclei, and FISH spots analyzed in nocodazole treatment experiments described in Figure 4.

Gene	Condition	n_{fibers}	n_{nuclei}	n_{spots}
Dmd	DMSO	8	36	928
	nocodazole	8	23	754
	washout	6	30	648
Gapdh	DMSO	9	37	93453
	nocodazole	8	36	82495
	washout	3	18	33382
Hist1h1c	DMSO	7	28	7801
	nocodazole	12	69	5658
	washout	4	24	5496
Hnrnpa2b1	DMSO	8	43	7274
	nocodazole	5	29	4357
	washout	5	32	4015
Myom1	DMSO	5	26	3163
	nocodazole	5	28	2983
	washout	5	20	2462
Polr2a	DMSO	5	26	1223
	nocodazole	5	26	1537
	washout	5	22	1616
Ttn	DMSO	11	62	43153
	nocodazole	8	45	25524
	washout	5	34	18009
Vcl	DMSO	5	26	2485
	nocodazole	5	24	1083
	washout	5	19	1160

Supplementary Table 3. Numbers of fibers, nuclei, and FISH spots analyzed in Actinomycin D treatment experiments described in Supplementary Figure 4.

Gene	Treatment time (hr)	n_{fibers}	n_{nuclei}	n_{spots}
Hnrnpa2b1	0	6	30	3434
Hnrnpa2b1	6	3	16	1324
Hnrnpa2b1	20	3	11	1049
Polr2a	0	3	10	372
Polr2a	6	3	16	162
Polr2a	20	3	15	93
Myom1	0	6	31	4323
Myom1	6	3	21	2801
Myom1	20	3	18	1708
Gapdh	0	3	12	22800
Gapdh	6	3	20	35334
Gapdh	20	3	13	14396
Hist1h1c	0	5	27	1558
Hist1h1c	6	3	16	541
Hist1h1c	20	3	19	362
Vcl	0	5	24	878
Vcl	6	3	17	386
Vcl	20	3	13	259
Ttn	0	3	13	14162
Ttn	6	4	19	10919
Ttn	20	3	9	5846
Dmd	0	3	13	231
Dmd	6	3	16	287
Dmd	20	3	15	129
