# Supplementary material for: Microtubule-based transport is essential to distribute RNA and nascent protein in skeletal muscle
Source: Nat Commun. 2021 Oct 27;12:6079. doi: 10.1038/s41467-021-26383-9 (PMC8551216; doi:10.1038/s41467-021-26383-9)
Supplement: Supplementary file 2 — Description of Additional Supplementary Files [file 41467_2021_26383_MOESM2_ESM.pdf]

Description of Additional Supplementary Files

File Name: Supplementary Movie 1
Description: **mRNAs Are Detected In Myofibers, at the Surface and in the Core, Related to Figure 1.** FISH of Ttn mRNA (green) and Hnrnpa2b1 mRNA (red) in isolated myofiber. Myonuclei labeled with DAPI (blue). Video shows 0.5 µm optical sections extending in 8 µm from the surface of a myofiber. Scale bar: 5 µm.

File Name: Supplementary Movie 2
Description: **The Microtubule Lattice Interweaves Throughout Sarcomeres of Skeletal Myofibers, Related to Figure 1.** Co-IF of tubulin protein (green) to label the microtubule network and telethonin protein (red) to label the sarcomere Z-disks in an isolated myofiber. Myonuclei labeled with DAPI (blue). Video shows 0.5 µm optical sections extending in 4 µm from the surface of a myofiber. Scale bars: 5 µm.

File Name: Supplementary Movie 3
Description: **mRNAs Upregulated During Myofiber Culture Localize Furthest from Progenitor Myonuclei Along the Longitudinal Poles, Related to Figure 3.** FISH of Hist1h1c mRNA in an isolated myofiber cultured for 18 hr with DMSO (control treatment, see Fig. S3A). Myonuclei labeled with DAPI (blue). Video shows 0.5 µm optical sections extending in 7 µm from the surface of a myofiber. Scale bar: 5 µm.

File Name: Supplementary Movie 4
Description: **MS2 Reporter RNAs in Live C2C12 Myotubes, Related to Figure 8.** Videos show five myotubes expressing MS2 labeled RNA (top) with RNA trajectories overlaid (bottom). Scale bars: 2 µm.

File Name: Supplementary Movie 5
Description: **Live C2C12 Myotubes Expressing MCP-Halo Without MS2 Reporter, Related to Figure 8.** Scale bars: 2 µm.

File Name: Supplementary Movie 6
Description: **Example Tracks from Each Category of RNA Motion, Related to Figure 8.** Particles from myotubes from Video S3 were categorized into one of four motion states (see Methods), two diffusive (“Low Mobility” and “High Mobility”) and two directed (“Crawling” and “Processive”). Scale bars: 0.5 µm.

File Name: Supplementary Movie 7
Description: **MS2 Reporter RNAs in Live C2C12 Myotubes Treated With Nocodazole, Related to Figure 8.** Videos show myotubes expressing MS2 labeled RNA acquired 10 min after treatment with 5 µg/mL nocodazole. Scale bars: 2 µm.

File Name: Supplementary Movie 8
Description: **RNPs Split and Merge in Myotubes, Related to Figure 8.** Zoomed regions of myotubes from Video S2 showing RNPs undergoing directed transport events in which they split from (first video), or split and merge with (second video) other RNPs. Scale bars: 0.5 µm.

File Name: Supplementary Movie 9
Description: **MS2 Reporter RNAs in Live C2C12 Myotubes Imaged for 50 min, Related to Figure 8.** Videos show five myotubes expressing MS2 labeled RNA (top) with RNA trajectories overlaid (bottom). Scale bars: 2 µm.

File Name: Supplementary Movie 10
Description: **Computational Simulation Confirms That Directed Transport Is Required to Disperse mRNA in Myofibers, Related to Figure 9.** 10 hr of simulated RNA motion in a segmentation of a real myofiber (see Fig. 1 and S1) in different configurations of diffusive and directed motion states. Videos show 2D projections of three-dimensional simulations. Simulated RNAs in "low-mobility" and "high-mobility" diffusive states are represented as blue and green dots, respectively. "Processive'' and "Crawling" transport events are shown as magenta and yellow line segments, respectively. Grey dots represent decayed RNAs. Nuclei shown in white. Scale bars: 10 µm.
