# Supplementary material for: Microtubule-based transport is essential to distribute RNA and nascent protein in skeletal muscle
Source: Nat Commun. 2021 Oct 27;12:6079. doi: 10.1038/s41467-021-26383-9 (PMC8551216; doi:10.1038/s41467-021-26383-9)
Supplement: Supplementary file 13 — Reporting Summary [file 41467_2021_26383_MOESM13_ESM.pdf]

nature research

Corresponding author(s): Eric T. Wang

Last updated by author(s): 9/7/2021

Reporting Summary

Nature Research wishes to improve the reproducibility of the work that we publish. This form provides structure for consistency and transparency in reporting. For further information on Nature Research policies, see our Editorial Policies and the Editorial Policy Checklist.

Statistics

For all statistical analyses, confirm that the following items are present in the figure legend, table legend, main text, or Methods section.

n/a	Confirmed	
☐	☒	The exact sample size (*n*) for each experimental group/condition, given as a discrete number and unit of measurement
☐	☒	A statement on whether measurements were taken from distinct samples or whether the same sample was measured repeatedly
☐	☒	The statistical test(s) used AND whether they are one- or two-sided<br>*Only common tests should be described solely by name; describe more complex techniques in the Methods section.*
☐	☒	A description of all covariates tested
☐	☒	A description of any assumptions or corrections, such as tests of normality and adjustment for multiple comparisons
☐	☒	A full description of the statistical parameters including central tendency (e.g. means) or other basic estimates (e.g. regression coefficient) AND variation (e.g. standard deviation) or associated estimates of uncertainty (e.g. confidence intervals)
☐	☒	For null hypothesis testing, the test statistic (e.g. *F*, *t*, *r*) with confidence intervals, effect sizes, degrees of freedom and *P* value noted<br>*Give P values as exact values whenever suitable.*
☒	☐	For Bayesian analysis, information on the choice of priors and Markov chain Monte Carlo settings
☒	☐	For hierarchical and complex designs, identification of the appropriate level for tests and full reporting of outcomes
☐	☒	Estimates of effect sizes (e.g. Cohen's *d*, Pearson's *r*), indicating how they were calculated

Our web collection on statistics for biologists contains articles on many of the points above.

Software and code

Policy information about availability of computer code

Data collection	Image collection was performed using Zen Black software (Zeiss, Version 14.0.15.201).
Data analysis	Airyscan processing was performed using Zen Black software (Zeiss, Version 14.0.15.201). Segmentation and quantification of all myofiber microscopy data was performed using python (version 3.7.6). Quantification of live cell myotube microscopy data was performed using ImageJ (version 2.1.0/1.53f). All plots were generated using python (version 3.7.6). Custom python code used in the study is available on GitHub (https://github.com/cpkelley94/muscle-FISH) (version 1.0.0) DOI: 10.5281/zenodo.5484423.

For manuscripts utilizing custom algorithms or software that are central to the research but not yet described in published literature, software must be made available to editors and reviewers. We strongly encourage code deposition in a community repository (e.g. GitHub). See the Nature Research guidelines for submitting code & software for further information.

Data

Policy information about availability of data

All manuscripts must include a data availability statement. This statement should provide the following information, where applicable:

- Accession codes, unique identifiers, or web links for publicly available datasets
- A list of figures that have associated raw data
- A description of any restrictions on data availability

Source data is provided for all figures in this study. Additional supporting data are available from the corresponding author upon reasonable request.

April 2020

Field-specific reporting

Please select the one below that is the best fit for your research. If you are not sure, read the appropriate sections before making your selection.

- [x] Life sciences
- [ ] Behavioural & social sciences
- [ ] Ecological, evolutionary & environmental sciences

For a reference copy of the document with all sections, see nature.com/documents/nr-reporting-summary-flat.pdf

Life sciences study design

All studies must disclose on these points even when the disclosure is negative.

Sample size	For all experiments we analyzed a sufficient number of myofibers or myotubes to observe clear distributions of all measurements for each RNA and condition combination analyzed. A minimum of n=3 cells were used in all cases. We did not perform any specific statistical tests to determine sample sizes.
Data exclusions	No data were excluded.
Replication	All experiments were repeated at least 3 times to confirm all results and all replication attempts were successful.
Randomization	In all myofiber experiments, myofibers were isolated from multiple mice of both sexes and distributed randomly across different conditions. In all myotube experiments, myotubes were plated onto identical dishes and allocated at random to different experimental groups.
Blinding	In all experiments, investigators were blinded to RNA identity and allocation of experimental conditions during microscopy data collection and analysis using a numbered sample key. Differences in expression levels between RNAs and obvious effects of experimental treatments interfered with the efficacy of blinding, however unbiased quantitative methods were used in all analyses.

Reporting for specific materials, systems and methods

We require information from authors about some types of materials, experimental systems and methods used in many studies. Here, indicate whether each material, system or method listed is relevant to your study. If you are not sure if a list item applies to your research, read the appropriate section before selecting a response.

Materials & experimental systems

n/a	Involved in the study	
[ ]	[x]	Antibodies
[ ]	[x]	Eukaryotic cell lines
[x]	[ ]	Palaeontology and archaeology
[ ]	[x]	Animals and other organisms
[x]	[ ]	Human research participants
[x]	[ ]	Clinical data
[x]	[ ]	Dual use research of concern

Methods

n/a	Involved in the study	
[x]	[ ]	ChIP-seq
[x]	[ ]	Flow cytometry
[x]	[ ]	MRI-based neuroimaging

Antibodies

Antibodies used	Polyclonal rabbit anti-Fxr1p (13194-1-AP, Proteintech; dilution: 1:200)<br>polyclonal rabbit anti-KIF1C (ab125903, Abcam; dilution: 1:500)<br>polyclonal rabbit anti-Mbnl1 (kindly gifted by Maury Swanson; dilution: 1:1000)<br>monoclonal rabbit anti-Telethonin (clone: EPR8375, ab133646, Abcam; dilution: 1:1000)<br>monoclonal mouse anti-TDP-43 (clone 3H8, ab104223, Abcam; dilution: 1:500)<br>monoclonal mouse anti-G3BP1 (clone 2F3, ab56574, Abcam; dilution: 1:1000)<br>monoclonal mouse anti-Alpha Tubulin (clone AA13, T8203, Sigma; dilution: 1:1000)<br>monoclonal mouse anti-Puromycin (clone 3RH11, EQ0001, Kerafast; dilution: 1:1000)<br>monoclonal mouse anti-Nuclear Pore Complex Proteins (clone MAB414, Abcam, ab24609; dilution: 1:1000)<br>polyclonal chicken anti-Alpha Tubulin (ab89984, abcam; dilution: 1:1000)
Validation	anti-Fxr1p KD validated in Cao et al. PMID: 30691465<br>anti-Kif1c validated on patients with nonsense mutations in Dor et. al. doi:10.1136/jmedgenet-2013-102012<br>anti-Mbnl1 KO validated in Kanadia et al. doi: 10.1073/pnas.0604970103<br>anti telethonin used according to abcam specifications and validated by immunofluorescence and comparison to previous published images of telethonin staining in skeletal muscle samples, for which it matched<br>anti-tdp43 KO validated by abcam<br>anti-G3BP1 validated in Matheny et al. doi: 10.1261/rna.078204.120 via autofluorescence before and after induction of stress granule formation and subsequent comparison to known distribution of G3BP1 signal (localized to stress granules)

anti-tubulin 157 citations on https://www.citeab.com/antibodies/2304942-t8203-anti-alpha-tubulin-antibody-mouse-monoclonal?des=077af1f794e8f08e
anti-puromycin validated by treatment with inhibitors of puromyclation followed by IF showing no signal
anti-nuclear pore complex proteins 134 citations on https://www.citeab.com/antibodies/770253-ab24609-anti-nup107-antibody-mab414?des=4c1461c6c6c6b816
anti-tubulin (chicken) validated by immunofluorescence and comparison to known microtubule structure

Eukaryotic cell lines

Policy information about cell lines

Cell line source(s)	C2C12 cell line from ATCC
Authentication	C2C12 cell line was authenticated via morphological assessment at the monocellular stage and assessed for fusion capability by differentiation and morphological assessment
Mycoplasma contamination	Cell lines were not tested for mycoplasma
Commonly misidentified lines (See ICLAC register)	No commonly misidentified cell lines were used in this study.

Animals and other organisms

Policy information about studies involving animals; ARRIVE guidelines recommended for reporting animal research

Laboratory animals	10 week old FVB/NJ mice of both sexes
Wild animals	No wild animals were used in this study.
Field-collected samples	No field collected samples were used in this study.
Ethics oversight	University of Florida IAUCUC and ACS

Note that full information on the approval of the study protocol must also be provided in the manuscript.
